# Sensing of H_2_O_2_-induced oxidative stress by the UPF factor complex is crucial for activation of *catalase-3* expression in *Neurospora*

**DOI:** 10.1371/journal.pgen.1010985

**Published:** 2023-10-16

**Authors:** Shuangjie Shen, Chengcheng Zhang, Yuanhao Meng, Guofei Cui, Ying Wang, Xiao Liu, Qun He

**Affiliations:** 1 MOA Key Laboratory of Soil Microbiology, College of Biological Sciences, China Agricultural University, Beijing, China; 2 State Key Laboratory of Mycology, Institute of Microbiology, Chinese Academy of Sciences, Beijing, China; 3 College of Life Sciences, University of the Chinese Academy of Sciences, Beijing, China; Oregon State University, UNITED STATES

## Abstract

UPF-1-UPF-2-UPF-3 complex-orchestrated nonsense-mediated mRNA decay (NMD) is a well-characterized eukaryotic cellular surveillance mechanism that not only degrades aberrant transcripts to protect the integrity of the transcriptome but also eliminates normal transcripts to facilitate appropriate cellular responses to physiological and environmental changes. Here, we describe the multifaceted regulatory roles of the *Neurospora crassa* UPF complex in *catalase-3* (*cat-3*) gene expression, which is essential for scavenging H_2_O_2_-induced oxidative stress. First, losing UPF proteins markedly slowed down the decay rate of *cat-3* mRNA. Second, UPF proteins indirectly attenuated the transcriptional activity of *cat-3* gene by boosting the decay of *cpc-1* and *ngf-1* mRNAs, which encode a well-studied transcription factor and a histone acetyltransferase, respectively. Further study showed that under oxidative stress condition, UPF proteins were degraded, followed by increased CPC-1 and NGF-1 activity, finally activating *cat-3* expression to resist oxidative stress. Together, our data illustrate a sophisticated regulatory network of the *cat-3* gene mediated by the UPF complex under physiological and H_2_O_2_-induced oxidative stress conditions.

## Author summary

The UPF complex-mediated NMD pathway is an evolutionarily conserved mRNA surveillance mechanism in eukaryotic cells, which degrades both mutated mRNAs to protect the transcriptome integrity and normal mRNAs to facilitate appropriate cellular responses. In fission yeast, mutant strains lacking UPF proteins are extremely sensitive to H_2_O_2_-induced oxidative stress. Here, we discovered a completely opposite H_2_O_2_-resistance mechanism in *upf* deletion mutants of the filamentous fungus *N*. *crassa*. Under physiological conditions, the *N*. *crassa* UPF complex inhibits the expression of the *cat-3* gene to maintain redox homeostasis. In the presence of H_2_O_2_, UPF proteins are degraded and thus allow for the expression of the *cat-3* gene, eventually ensuring adequate catalase activity to deal with oxidative stress. Our results do not only open up the post-transcriptional regulatory mechanism of catalase gene, but also identify the UPF complex as key node in response to oxidative stress.

## Introduction

The precise expression of protein-coding genes is crucial for the execution of physiological processes and the maintenance of cellular homeostasis. In general, gene expression is balanced by the transcription, processing, degradation and translation of the corresponding mRNA. The multistep nature of gene expression, while enabling multilevel regulation, introduces more opportunities for error. To maintain the fidelity of gene expression, mRNAs are generally inspected for errors to prevent cells from producing potentially deleterious proteins in bulk [1].

Nonsense-mediated mRNA decay (NMD) is an evolutionarily conserved mRNA surveillance mechanism that identifies and degrades aberrant mRNAs harboring premature termination codon (PTC) from the transcriptome through intricate steps [1–3]. PTCs arise from many sources, including genetic variation, somatic mutation, unfaithful RNA transcription or splicing and incorrect choice of translation start site. Such PTC-containing mRNAs constitute ~30% of all known disease-associated mutations owing to the generation of nonfunctional or even dominant-negative proteins [4]. The three conserved factors, UPF1, UPF2 and UPF3, form a complex and constitute the core NMD component in eukaryotes [5–11]. Among them, RNA helicase UPF1 is considered to be the master regulator of the NMD pathway because it undergoes most steps from recognition of the PTC-containing mRNAs until their degradation. The helicase activity of UPF1 for specific PTC-containing mRNAs depends on its ATPase hub and phosphorylation by the SMG1 kinase [12–16]. UPF1 and its associated kinase, SMG1, directly bind to the eukaryotic release factors eRF1 and eRF3 to form the surveillance complex (SURF) in the vicinity of the PTC. Subsequent interactions of SURF with UPF2, UPF3 and exon junction complex (EJC) downstream of the PTC trigger the formation of the decay-inducing complex (DECID), resulting in SMG1-mediated UPF1 phosphorylation and dissociation of eRF1 and eRF3 [12, 17, 18]. Phosphorylated UPF1 further recruits different phospho-binding factors to initiate mRNA decay: 1) recruitment of the endonuclease SMG6 to catalyze PTC-proximal mRNA cleavage, ultimately producing 5’ and 3’ cleavage fragments that are degraded by general cellular exonucleases [19–23]; and 2) recruitment of the SMG5-SMG7 heterodimer to bridge the interaction with the CCR4-NOT deadenylase complex, thereby shortening the poly(A) tail to stimulate mRNA decapping by the general decapping complex and 5’-3’ degradation by the exonuclease XRN1 [24, 25].

Interestingly, eukaryotic cells also exploit NMD to control the quantities of normally occurring mRNAs in diverse physiological conditions. Approximately 10% of normal transcripts are potential substrates for NMD-mediated degradation, which contain specific sequence features that can trigger NMD, including upstream open reading frames (uORFs), long 3’UTR or introns located in 3’UTR region [8, 10, 26–31]. Although the molecular mechanisms of NMD for normal transcripts remain unclear, the degradations of transcripts carrying different sequence features probably depend on different mechanisms. Studies in *N*. *crassa* have shown that the NMD pathway induced by spliced 3’-UTR introns requires EJC factors, but uORFs or long 3’-UTR feature triggered NMD occurs independently of the EJC [8, 10, 31]. NMD activity itself varies between cell types, tissues, and developmental stages and a number of post-transcriptional regulatory mechanisms modulating NMD activity have been identified [32]. Through dynamically adjusting the abundance of the transcriptome, NMD regulates numerous critical physiological processes during differentiation, development and death as well as cellular stress responses in eukaryotes [8, 9, 31, 33–38]. For example, the long 3’-UTR-containing *casein kinase I* (*ck-1a*) transcript is subject to mutually antagonistic regulation by NMD and an RNA-binding protein, PRD-2, to control its optimal gene expression and maintain a normal circadian period in *N*. *crassa*. This finding has led to the view that modulations in NMD magnitude alter the transcriptome to achieve specific biological outcomes [10].

Eukaryotic cells are unavoidably exposed to diverse stress stimuli during growth and development. Among them, oxidative stress ubiquitously exists and severely threatens normal physiological activities of aerobic organisms. Oxidative stress generally arises from the homeostasis disturbance between the excessive production of reactive oxygen species (ROS) and the insufficient ROS scavenging capacity in cells [39]. ROS refer to unstable and reactive oxygen-containing molecules, such as superoxide anion (O2^•−^), hydrogen peroxide (H_2_O_2_), hydroxyl radical (•OH) and singlet oxygen (^1^O_2_) [39, 40]. If left unchecked, persistent oxidative stress will induce cellular dysfunction, diseases occurrence and even death through irreversibly impairing cellular biomacromolecules, including lipids, proteins and nucleic acids [41–44]. To eliminate the accumulation of toxic ROS, aerobic organisms have evolved various ROS scavengers such as catalase (CAT) that decomposes H_2_O_2_ into water and oxygen [45–50]. Catalase is highly conserved in aerobic organisms [51, 52]. The filamentous fungus *N*. *crassa* possesses three catalases, CAT-1, CAT-2 and CAT-3, which are expressed in the different phases of its asexual life cycle [50, 53, 54]. CAT-1 is mainly accumulated in conidia, CAT-2 is generally produced in aerial hyphae and conidia, whereas CAT-3 activities are gradually increased during the exponential growth phase of mycelia [55–57]. CAT-3 is the predominant and irreplaceable catalase in resisting H_2_O_2_-induced oxidative stress [55]. Therefore, it is necessary to comprehensively dissect the regulatory network of *cat-3* gene expression at physiological conditions and oxidative stress. Earlier studies reported that the *N*. *crassa* transcription factor CPC-1 is required for the transcriptional activation of cycloheximide-inducible *laccase* (NCU04528) gene, which belongs to the multi-copper oxidases and catalyzes the oxidation of organic substrates with the concomitant reduction of molecular oxygen to water [58]. Consistently, previous studies showed that CPC-1 coordinates with histone acetyltransferase NGF-1, the homologue of the yeast Gcn5p, to positively regulate *cat-3* gene expression in response to H_2_O_2_-induced oxidative stress [59–61]. However, post-transcriptional regulation of *cat-3* expression remains a relatively untouched area. Previous studies in fission yeast revealed that mutant strains lacking *upf1*, *upf2* or *upf3* are extremely sensitive to H_2_O_2_-induced oxidative stress, suggesting that UPF proteins are required for normal resistance to oxidative stress in fission yeast [36, 38]. Although several studies about UPF functions have been reported in *N*. *crassa*, it is still unknown whether the *N*. *crassa* UPF proteins participate in the response to oxidative stress.

Here, we demonstrated that unlike in fission yeast, deletion of *N*. *crassa* UPF proteins significantly upregulated *cat-3* gene expression and therefore endowed strains with strong resistance to H_2_O_2_-induced oxidative stress. After H_2_O_2_ treatment, UPF proteins were degraded, consequently releasing *cat-3* gene expression to deal with oxidative stress. Our results demonstrate the multilevel regulatory roles of *N*. *crassa* UPF complex in *cat-3* gene expression, which is crucial for responding to H_2_O_2_-induced oxidative stress.

## Results

### 1. *upf* mutants are resistant to H_2_O_2_-induced oxidative stress through upregulating *cat-3* gene expression in *N*. *crassa*

**Fig 1:**
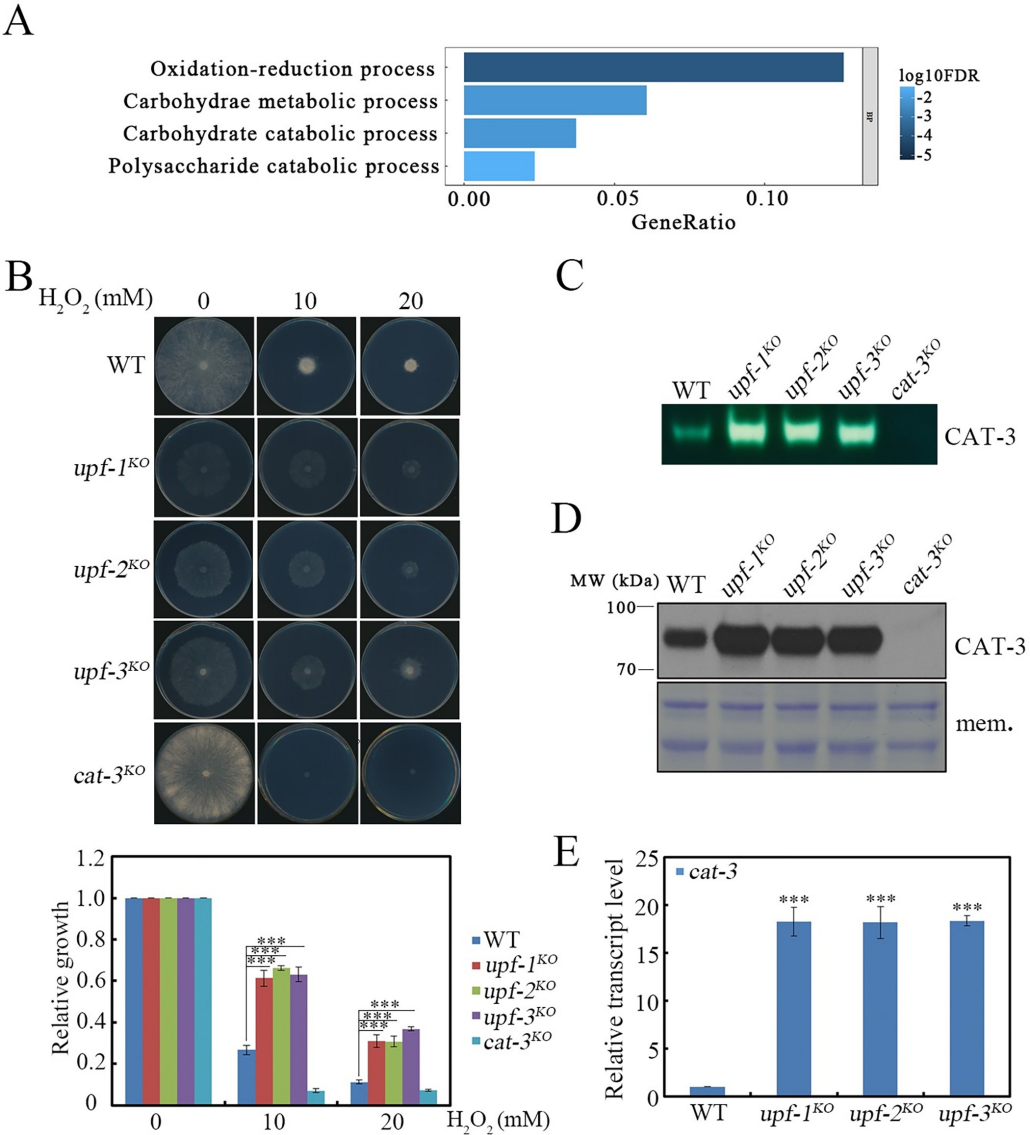
UPF proteins repress *cat-3* gene expression. (A) Distribution of the GO categories of genes upregulated in the *upf-1*^*KO*^ strain revealed by RNA-seq analysis. Genes showing expression with fold change≥2 were analyzed. BP: Biological Process; FDR: False Discovery Rate. (B) Plate assays analyzing mycelial growth of WT, *upf-1*^*KO*^, *upf-2*^*KO*^ and *upf-3*^*KO*^ strains under different H_2_O_2_ concentrations (upper figure). Relative growth of mycelia was measured (lower figure). (C) In-gel assays showing the CAT-3 activities of WT, *upf-1*^*KO*^, *upf-2*^*KO*^ and *upf-3*^*KO*^ strains. (D) Western blot showing the levels of CAT-3 protein in WT, *upf-1*^*KO*^, *upf-2*^*KO*^ and *upf-3*^*KO*^ strains. The membrane stained by Coomassie blue represented the total protein in each sample and served as the loading control. (E) RT-qPCR assays showing the levels of *cat-3* mRNA in WT, *upf-1*^*KO*^, *upf-2*^*KO*^ and *upf-3*^*KO*^ strains. Each experiment was performed at least three times independently. Error bars indicate S.D. (*n* = 3). *P **<** 0.05; **P **<** 0.01; ***P **<** 0.001. Unpaired Student’s *t* test was used. The *cat-3*^*KO*^ strain was used as the negative control in (B) (C) (D).

UPF proteins, the core components of the NMD pathway, are necessary for normal resistance to H_2_O_2_-induced oxidative stress in fission yeast [36, 38]. To determine whether *N*. *crassa* UPF proteins function as they do in fission yeast, we first performed RNA-seq and GO analysis in wild-type (WT) and *upf-1* deletion mutant (S1 Table). Unexpectedly, redox-related pathways were significantly upregulated in the *upf-1* deletion mutant compared to WT strain (Fig 1A), suggesting that UPF-1 represses redox processes in *N*. *crassa*. Next, we examined the growth phenotypes and H_2_O_2_ sensitivities of the deletion strains of UPF-1 (NCU04242), UPF-2 (NCU05267) or UPF-3 (NCU03435). As shown in Figs 1B and S1A, the three *upf* mutants grew slowly in physiological conditions, they were all strongly resistant to H_2_O_2_-induced oxidative stress compared to the WT strain [8, 62]. Considering that CAT-3 protein is the predominant catalase for scavenging H_2_O_2_-induced oxidative stress [55, 56], we measured CAT-3 activities in the WT strain and *upf* mutants by in-gel assays. As expected, CAT-3 activities were obviously increased in the *upf-1*^*KO*^, *upf-2*^*KO*^ and *upf-3*^*KO*^ strains compared to those of the WT strain (Fig 1C), indicating that the H_2_O_2_-resistant phenotypes observed in the *upf* mutants were due to the significantly elevated CAT-3 activities. To explore the causes of elevated CAT-3 activities in *upf* mutants, we measured the expression levels of *cat-3* gene. Western blot and RT-qPCR results showed that the levels of CAT-3 protein (Fig 1D) and *cat-3* mRNA (Fig 1E) in three *upf* mutants remarkably increased compared to those found in the WT strain. In addition, a large halo of catalases activity was much brighter in the *upf* ^*KO*^ strains compared to those in the WT and *cat-3*^*KO*^ strains (S1B Fig). To exclude the effect of *ras-1*^*bd*^ mutation on UPF mediated repression of *cat-3* gene expression, we examined the CAT-3 activity and protein level in the FGSC 4200 (ORS-SL6a) WT and *upf* ^*KO*^ (*nbd*) strains. Consistent with those of the *ras-1*^*bd*^ stains, CAT-3 activities and protein levels also greatly increased in *upf* ^*KO*^ (*nbd*) strains compared to those of the WT strain (S1C and S1D Fig).

**Fig 2:**
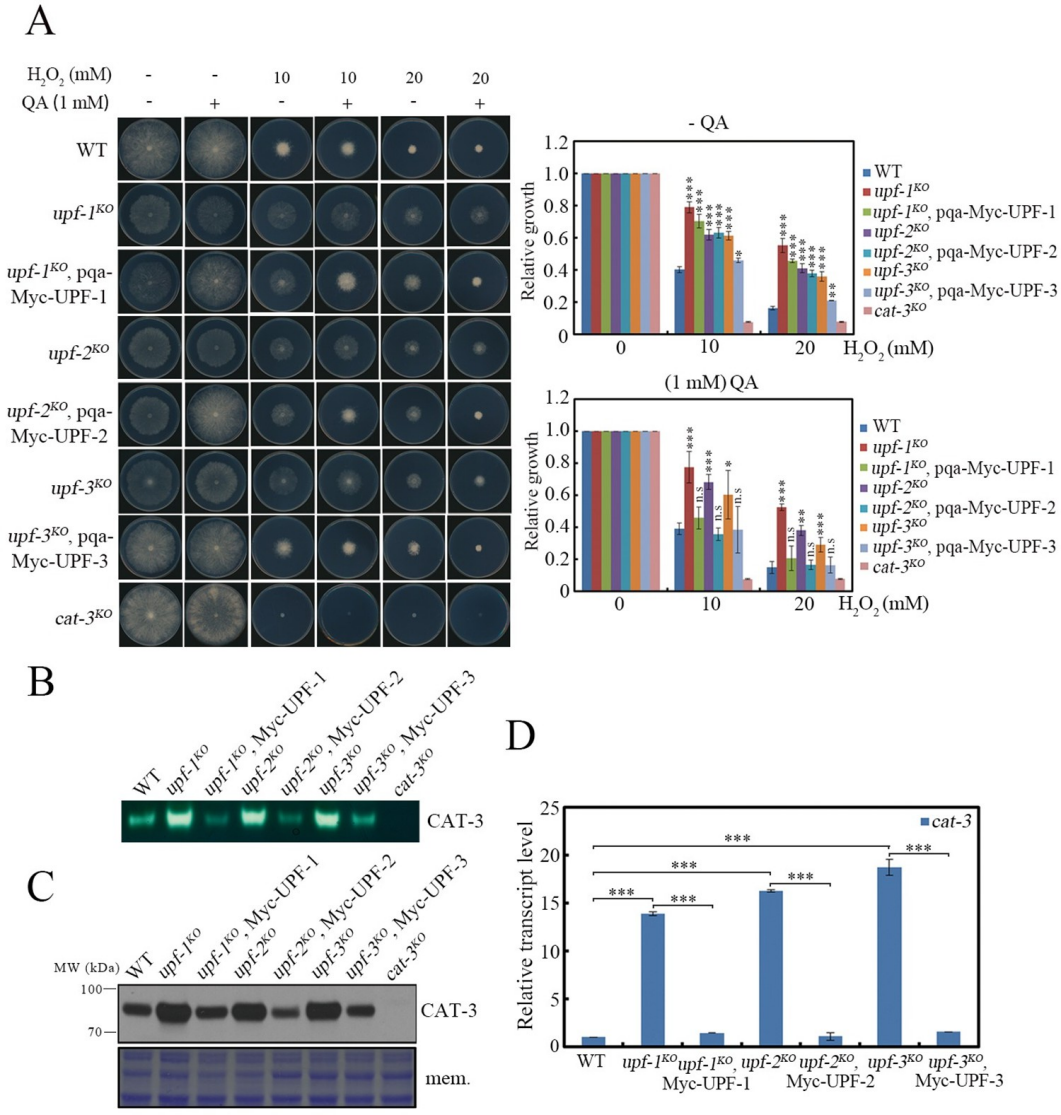
Ectopic expression of Myc-tagged UPF-1, UPF-2 or UPF-3 in the corresponding *upf* mutant can rescue *cat-3* gene regulation. (A) Plate assays analyzing mycelial growth (left) and relative growth (right) of WT, *upf-1*^*KO*^, *upf-2*^*KO*^, *upf-3*^*KO*^ strains and corresponding UPF-1, UPF-2, UPF-3 transformants driven by the *qa-2* promoter under different H_2_O_2_ concentrations. Quinic acid (QA) was used to induce the *qa-2* promoter. (B) In-gel assays showing the CAT-3 activities of WT, *upf-1*^*KO*^, *upf-2*^*KO*^, *upf-3*^*KO*^ strains and corresponding UPF-1, UPF-2, UPF-3 transformants. (C) Western blot showing the levels of CAT-3 protein in WT, *upf-1*^*KO*^, *upf-2*^*KO*^, *upf-3*^*KO*^ strains and corresponding UPF-1, UPF-2, UPF-3 transformants. The membrane stained by Coomassie blue represented the total protein in each sample and served as the loading control. (D) RT-qPCR assays showing the levels of *cat-3* mRNA in WT, *upf-1*^*KO*^, *upf-2*^*KO*^, *upf-3*^*KO*^ strains and corresponding UPF-1, UPF-2, UPF-3 transformants. Error bars indicate S.D. (*n* = 3). *P **<** 0.05; **P **<** 0.01; ***P **<** 0.001. Unpaired Student’s *t* test was used. The *cat-3*^*KO*^ strain was used as the negative control in (A) (B) (C).

To further confirm the role of UPF proteins in the H_2_O_2_-resistant phenotype in *N*. *crassa*, three plasmids encoding Myc-tagged UPF-1, UPF-2, or UPF-3 driven by the quinic acid (QA)-inducible promoter of *qa-2* gene were transformed into the *his-3* locus of each corresponding *upf* mutant. As shown in Fig 2A, ectopic expression of Myc-tagged UPF-1, UPF-2 or UPF-3 largely rescued the growth defects and H_2_O_2_ sensitivities of each corresponding *upf* mutant to those of the WT strain in the presence of QA (10^−3^ M), indicating that the observed phenotypes of these mutants were indeed due to the absence of each corresponding UPF protein. Consistent with the rescued phenotypes, the levels of CAT-3 activities, CAT-3 protein and *cat-3* mRNA in the *upf* complementary strains were also restored to those of the WT strain (Fig 2B–2D). Together, these results demonstrate that UPF proteins are the key regulators for the proper response to oxidative stress and *cat-3* gene expression in *N*. *crassa*.

We further constructed the *upf-1*^*KO*^ *upf-2*^*KO*^, *upf-1*^*KO*^ *upf-3*^*KO*^, and *upf-2*^*KO*^ *upf-3*^*KO*^ double mutant strains. As shown in S2A Fig, the growth defects and H_2_O_2_-resistant phenotypes of these three double mutants were all similar to those of each *upf* single mutant rather than exhibiting additive phenotypes, suggesting that the three UPF proteins function in the same pathway to regulate growth and resistance to H_2_O_2_-induced oxidative stress. Consistent with these observed phenotypes, the CAT-3 activities and CAT-3 protein levels in these double mutants were similar to the elevated levels in each single mutant but not in WT strains (S2B and S2C Fig). Together, these results demonstrate that UPF proteins regulate *cat-3* gene expression through the same pathway.

### 2. Conserved regions of UPF-1 and UPF-2 are required for suppression of *cat-3* expression

**Fig 3:**
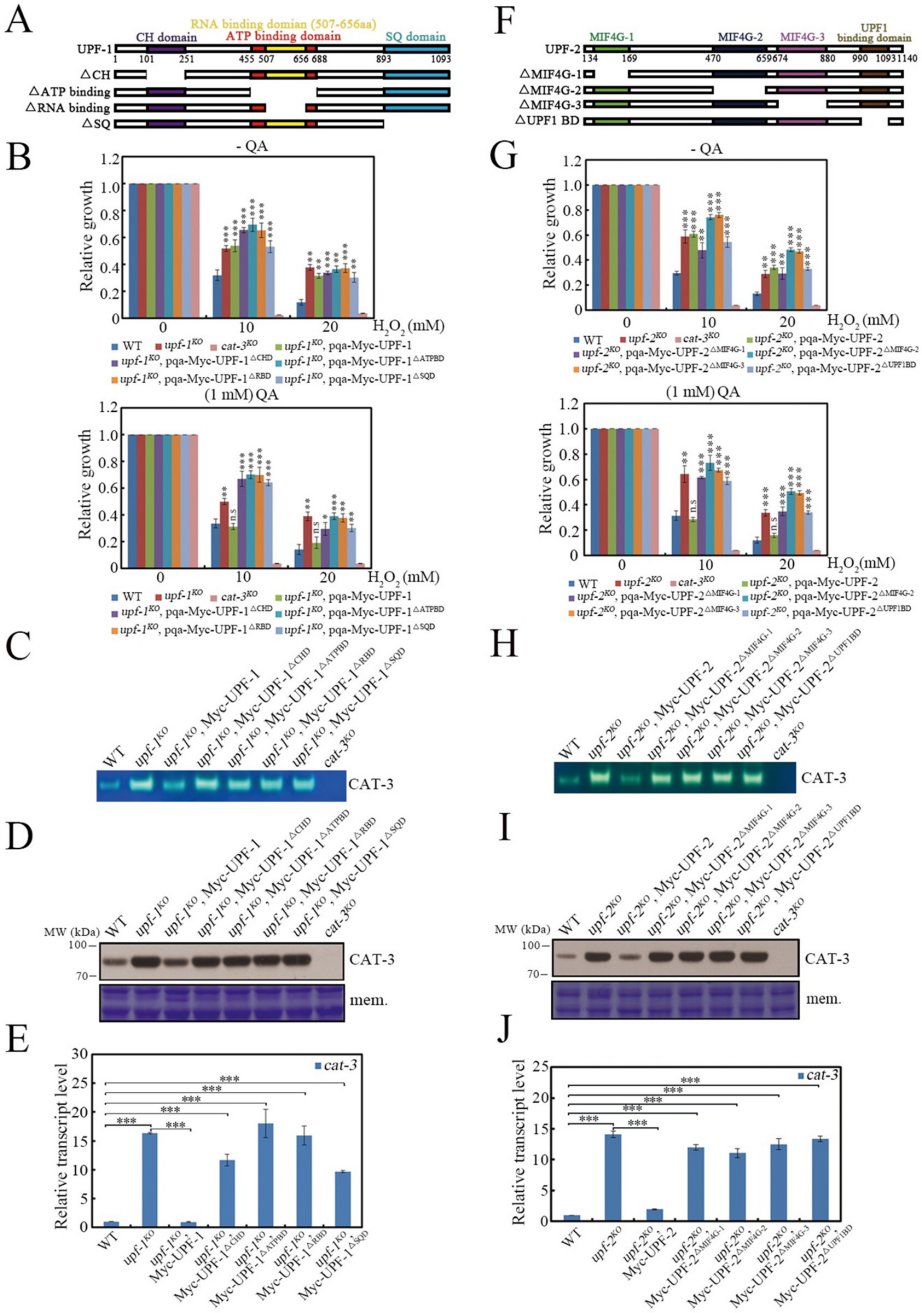
Conserved domains of UPF proteins are required for suppression of *cat-3* gene. (A and F) Schematic diagrams depicting the conserved domains of *N*. *crassa* UPF-1 (A) and UPF-2 (F) proteins as well as corresponding exogenous deletion mutants. The positions of CH (dark purple), ATP binding (red), RNA binding (yellow) and SQ (light blue) domains in UPF-1 as well as MIF4G-1 (green), MIF4G-2 (dark blue), MIF4G-3 (light purple) and UPF1 binding (brown) domains in UPF-2 are indicated. (B and G) Relative growth of the different deletion strains across UPF-1 (B) or UPF-2 (G) coding region at exogenous locus driven by *qa-2* promoter under different H_2_O_2_ concentrations. Quinic acid (QA) was used to induce the *qa-2* promoter. (C and H) In-gel assays showing the CAT-3 activities of the different deletion strains across UPF-1 (C) or UPF-2 (H) coding region at exogenous locus. (D and I) Western blot showing the levels of CAT-3 protein in the different deletion strains across UPF-1 (D) or UPF-2 (I) coding region at exogenous locus. The membrane stained by Coomassie blue represented the total protein in each sample and served as the loading control. (E and J) RT-qPCR assays showing the levels of *cat-3* mRNA in the different deletion strains across UPF-1 (E) or UPF-2 (J) coding region at exogenous locus. Error bars indicate S.D. (*n* = 3). *P **<** 0.05; **P **<** 0.01; ***P **<** 0.001. Unpaired Student’s *t* test was used. The *cat-3*^*KO*^ strain was used as the negative control in (B) (C) (D) (G) (H) (I).

Homologous sequence alignment revealed that UPF-1 is highly conserved in eukaryotes (S3A Fig) [8, 63, 64]. UPF1 contains a conserved helicase core, which exhibits ATPase and RNA binding activities through its internal ATP binding domain and RNA binding domain, respectively [65]. The helicase core is surrounded by an N-terminal cysteine–histidine rich region (CH domain) and a C-terminal serine-glutamine clusters (SQ domain). The CH domain binds to the C-terminal region of UPF2 to form the UPF surveillance complex, which is required for the NMD pathway [64]. On the other hand, the CH domain contains a putative RING domain that is structurally similar to the RING domain of known RING E3 ubiquitin ligases, which is responsible for UPF1’s protein degradation activity [66, 67]. The SQ domain contains numerous serine residues that are phosphorylated by SMG-1 to drive UPF1 function [63]. To characterize which region of UPF-1 functions in suppressing *cat-3* expression, we generated a series of deletion constructs across the UPF-1 coding region, including Myc-UPF-1^△CHD^, Myc-UPF-1^△ATPBD^, Myc-UPF-1^△RBD^ and Myc-UPF-1^△SQD^ (Fig 3A). All of the constructs were driven by the *qa-2* promoter and inserted into the *his-3* locus of the *upf-1* mutant. Western blot analysis showed that each short deletion had minor effect on the expression of the Myc-tagged UPF-1 protein (S3B Fig). As shown in Figs 3B and S3C, QA-induced ectopic expression of Myc-UPF-1 largely restored the growth defects and H_2_O_2_ sensitivities of the *upf-1* mutant to those of the WT strain in the presence of QA (10^−3^ M), however, expression of each Myc-UPF-1 domain deletion protein failed to complement the growth defects and H_2_O_2_ sensitivities in the presence of QA. Similarly, expression of WT UPF-1, but not the mutated version of UPF-1 suppressed the high levels of CAT-3 activities, CAT-3 protein and *cat-3* mRNA in the *upf-1* mutant to the WT levels (Fig 3C–3E).

As the largest component of the UPF complex, UPF-2 is also relatively conserved in eukaryotes (S4A Fig) [8, 68, 69]. UPF2 comprises three tandem MIF4G (Middle domain of eIF4G) domains and a C-terminal UPF1 binding domain. MIF4G-1 and MIF4G-2 are important for recruitment of UPF2 to PTC-containing mRNAs through an unknown mechanism [70]. MIF4G-3 is the key module for NMD pathway because it interacts with the central RNA recognition motif (RRM) of UPF3. Thus, UPF2 is considered as the scaffold protein that bridges UPF1 and UPF3 [71]. We made the constructs Myc-UPF-2^△MIF4G-1^, Myc-UPF-2^△MIF4G-2^, Myc-UPF-2^△MIF4G-3^, and Myc-UPF-2^△UPF1BD^ to determine their roles in repressing *cat-3* expression in the *upf-2* mutant (Fig 3F). Western blot analysis showed that each short deletion had minor effect on the expression of the Myc-tagged UPF-2 protein (S4B Fig). Phenotype analysis and molecular examination showed that expression of Myc-UPF-2, but not the mutant Myc-UPF-2 proteins, largely rescued the H_2_O_2_ sensitivities (Figs 3G and S4C) by restoring CAT-3 activities, CAT-3 proteins and *cat-3* mRNA levels to WT levels in the presence of QA (Fig 3H–3J). Together, these results indicate that conserved regions of UPF-1 and UPF-2 proteins are required for suppression of *cat-3* expression.

### 3. Loss of UPF proteins simultaneously affects the degradation and synthesis of *cat-3* transcripts

**Fig 4:**
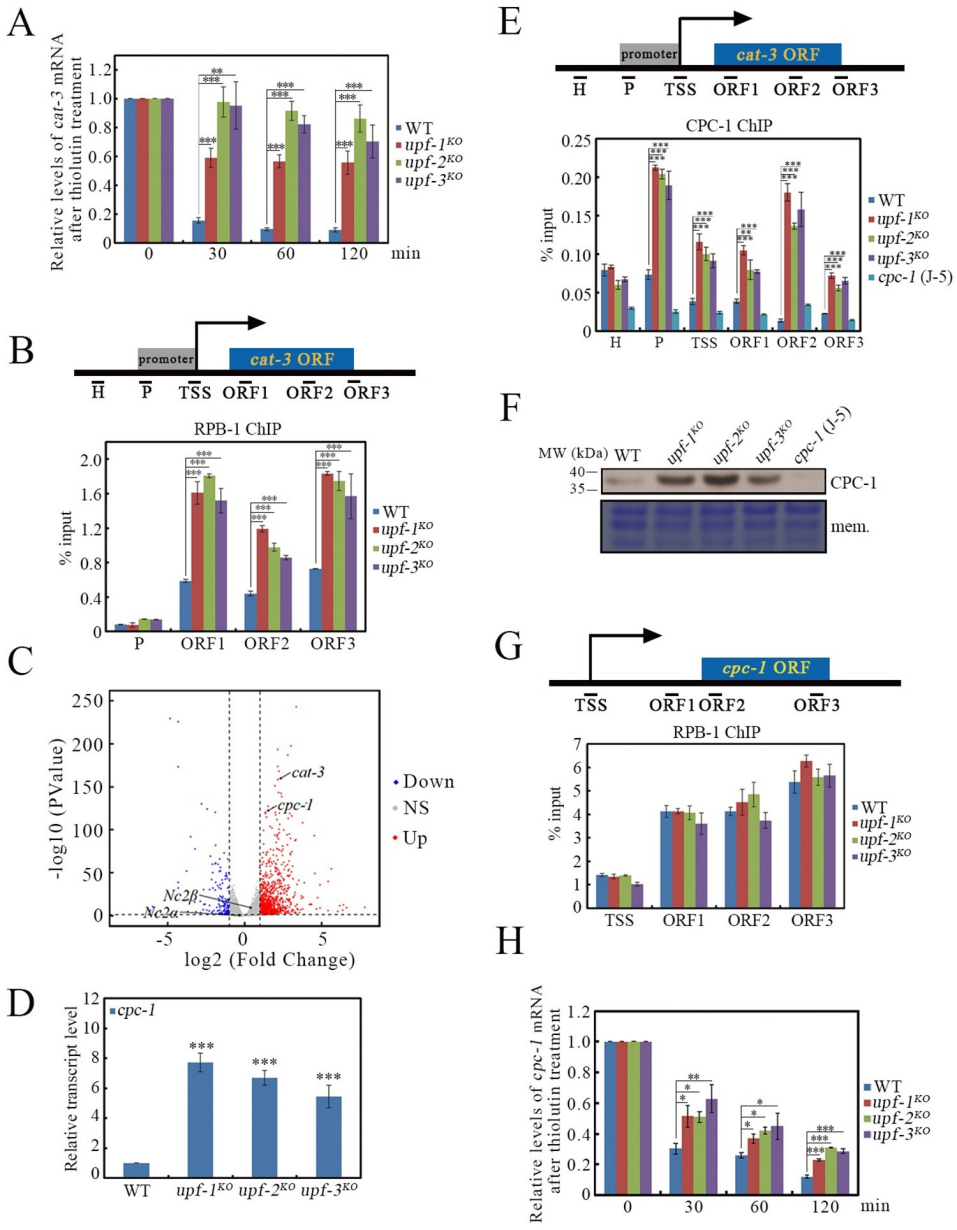
Loss of UPF proteins directly decreases the degradation of *cat-3* mRNA and indirectly increases *cat-3* gene transcription through elevating *cpc-1* mRNA stability. (A) RT-qPCR assays showing the relative degradation ratio of *cat-3* mRNA in WT, *upf-1*^*KO*^, *upf-2*^*KO*^ and *upf-3*^*KO*^ strains after the addition of thiolutin, respectively. (B) ChIP assays showing the binding levels of RPB-1 at *cat-3* gene locus in WT, *upf-1*^*KO*^, *upf-2*^*KO*^ and *upf-3*^*KO*^ strains. Primer pairs from upstream heterochromatin to ORF3 under the schematic diagram of *cat-3* gene locus indicate the regions detected by ChIP-qPCR. H, heterochromatin; P, promoter; TSS, transcription start site; ORF, open reading frame. (C) Volcano Plots of differential expressed genes (DEGs) between WT and *upf-1*^*KO*^ strains. The downregulated genes were labeled in blue, the upregulated genes were labeled in red, the gray areas show genes that are expressed at essentially the same level. NS, no significant. (D) RT-qPCR assays showing the levels of *cpc-1* mRNA in WT, *upf-1*^*KO*^, *upf-2*^*KO*^ and *upf-3*^*KO*^ strains. (E) ChIP assays showing the binding levels of CPC-1 at *cat-3* gene locus in WT, *upf-1*^*KO*^, *upf-2*^*KO*^ and *upf-3*^*KO*^ strains. The *cpc-1* (J-5) strain was used as the negative control. (F) Western blot showing the levels of CPC-1 protein in WT, *upf-1*^*KO*^, *upf-2*^*KO*^ and *upf-3*^*KO*^ strains. The membrane stained by Coomassie blue represented the total protein in each sample and served as the loading control. The *cpc-1* (J-5) strain was used as the negative control. (G) ChIP assays showing the binding levels of RPB-1 at *cpc-1* gene locus in WT, *upf-1*^*KO*^, *upf-2*^*KO*^ and *upf-3*^*KO*^ strains. TSS, transcription start site; ORF1-3, open reading frame1-3. (H) RT-qPCR assays showing the relative degradation ratio of *cpc-1* mRNA in WT, *upf-1*^*KO*^, *upf-2*^*KO*^ and *upf-3*^*KO*^ strains after the addition of thiolutin, respectively. Error bars indicate S.D. (*n* = 3). *P **<** 0.05; **P **<** 0.01; ***P **<** 0.001. Unpaired Student’s *t* test was used.

Although the targeted mechanisms of NMD for normal transcripts remain unclear, some specific features in normal mRNAs have been proven to elicit their decay through the NMD pathway, such as upstream open reading frames (uORFs) in 5’ UTR, long 3’UTR or introns in 3’UTR [1, 8–10, 62, 72]. *N*. *crassa cat-3* gene contains four uORFs which potentially encode peptides that are 21, 16, 30, or 92 amino acids in length. These four uORFs overlap with each other and the fourth uORF overlaps with the *cat-3* CDS in an out-of-frame manner (S5A Fig). Considering uORFs are signatures for NMD targets, we hypothesized that UPF proteins might regulate the *cat-3* transcripts stability. To test this hypothesis, we examined the degradation rate of *cat-3* mRNA after the addition of the mRNA synthesis inhibitor thiolutin. As shown in Fig 4A, *cat-3* mRNA stability in the *upf* mutants markedly increased compared to that in the WT strain, indicating that *cat-3* mRNA is a potential target of the UPF complex.

We next tested whether the deletion of UPF proteins affects *cat-3* gene transcription. To this end, we first performed ChIP assay to examine the enrichment of RPB-1, the largest subunit of RNAPII, at the *cat-3* gene locus in the WT and *upf* mutant strains. As shown in Fig 4B, the enrichment of RPB-1 at *cat-3* locus two-to-three-fold increased in the *upf* mutants compared to those of the WT strain, suggesting that the transcriptional activity of *cat-3* gene is significantly upregulated in the *upf* mutants. Previous studies identified negative cofactor 2 (NC2), including two subunits NC2α and NC2β, and the cross-pathway control transcription factor CPC-1 as key transcriptional activators for *cat-3* expression in *N*. *crassa* [59, 73–75]. RNA-seq assay and thiolutin treatment showed that *upf-1* deletion did not affect *Nc2β* mRNA stability compared with WT (Figs 4C and S5B). However, RNA-seq results demonstrated that only *cpc-1* transcripts were significantly elevated in the *upf-1* mutant (Fig 4C) (S2 Table). Previous studies have identified two upstream open reading frames located in the leading segment of the *cpc-1* coding region (S5C Fig) [31, 76]. ATF4 and GCN4, the functional homologous proteins of CPC-1 in humans and yeast, which contain uORFs, have been reported to be NMD targets [3, 77–79]. RT-qPCR assay further confirmed the significant upregulation of *cpc-1* mRNA in *N*. *crassa upf* mutant (Fig 4D). We therefore determined the enrichment of CPC-1 at *cat-3* gene locus in the *upf* mutants. As expected, deletion of UPF proteins markedly increased the enrichment of CPC-1 at the *cat-3* locus compared to that of the WT strain (Fig 4E). To explore the detailed reasons of increased CPC-1 binding, we measured the levels of CPC-1 proteins in the *upf* mutants. Consistent with the elevated CPC-1 enrichment, the levels of CPC-1 protein were observably higher in the *upf* mutants compared to those in the WT strain (Fig 4F). However, the degradation rate of CPC-1 proteins in the *upf-1*^*KO*^ mutant was comparable to that in the WT strain (S5D Fig), implying that increased levels of CPC-1 protein in the *upf* mutants might be attributed to the dysregulation of the *cpc-1* mRNA. To determine whether the elevated *cpc-1* mRNA levels are due to the increased expression of *cpc-1* gene in mutants, we checked the transcriptional activity of *cpc-1* in *upf* mutants. ChIP assay using an RPB-1-specific antibody revealed that loss of the UPF proteins had no effect on RPB-1 enrichment at *cpc-1* gene locus (Fig 4G). Instead, the stability of *cpc-1* mRNA in the *upf* mutants was markedly improved compared to that of the WT strain (Fig 4H). Taken together, these results indicate that UPF proteins target *N*. *crassa cpc-1* mRNA for its decay under physiological conditions.

**Fig 5:**
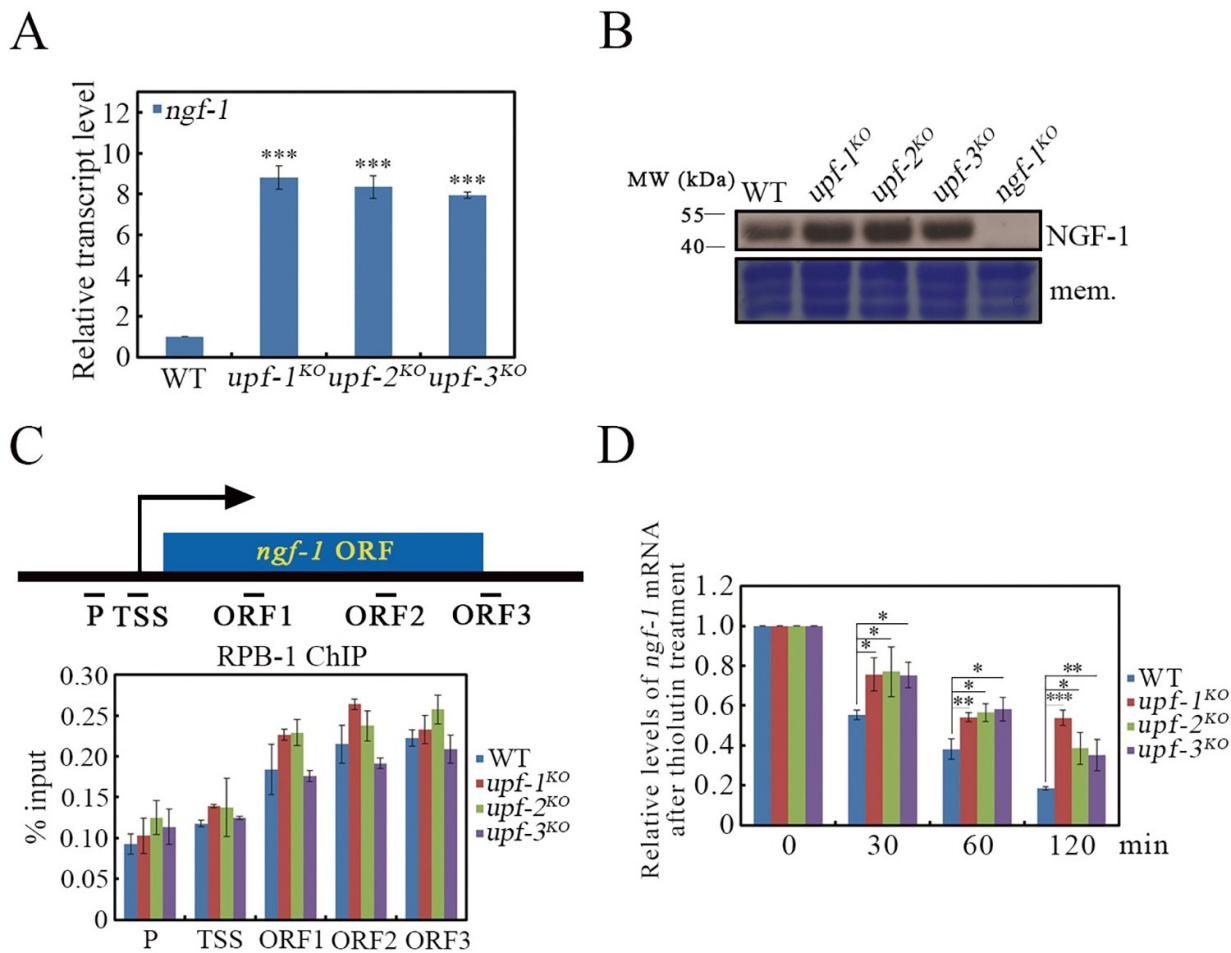
Loss of UPF proteins indirectly increases *cat-3* gene transcription through elevating *ngf-1* mRNA stability. (A) RT-qPCR assays showing the levels of *ngf-1* mRNA in WT, *upf-1*^*KO*^, *upf-2*^*KO*^ and *upf-3*^*KO*^ strains. (B) Western blot showing the levels of NGF-1 protein in WT, *upf-1*^*KO*^, *upf-2*^*KO*^ and *upf-3*^*KO*^ strains. The membrane stained by Coomassie blue represented the total protein in each sample and served as the loading control. The *ngf-1*^*KO*^ strain was used as the negative control. (C) ChIP assays showing the binding levels of RPB-1 at *ngf-1* gene locus in WT, *upf-1*^*KO*^, *upf-2*^*KO*^ and *upf-3*^*KO*^ strains. P, promoter; TSS, transcription start site; ORF1-3, open reading frame1-3. (D) RT-qPCR assays showing the relative degradation ratio of *ngf-1* mRNA in WT, *upf-1*^*KO*^, *upf-2*^*KO*^ and *upf-3*^*KO*^ strains after the addition of thiolutin, respectively. Error bars indicate S.D. (*n* = 3). *P **<** 0.05; **P **<** 0.01; ***P **<** 0.001. Unpaired Student’s *t* test was used.

Our previous study showed that CPC-1 coordinates with the histone acetyltransferase NGF-1 to regulate *cat-3* expression in *N*. *crassa* [59]. As shown in S6A Fig, the *ngf-1* transcript contains a long 3’UTR (1551 bp) that is a potential feature for NMD targeting. To test whether UPF proteins regulate the *ngf-1* gene in the same manner as the *cpc-1* gene, we generated a NGF-1 specific antibody (S6B Fig) and measured NGF-1 protein as well as *ngf-1* mRNA levels in the *upf* mutants. Similar to *cpc-1*, *ngf-1* also had significantly increased mRNA levels in the *upf* mutants (Fig 5A). As expected, loss of UPF proteins resulted in elevated levels of NGF-1 protein (Fig 5B), but had little impact on its stability (S6C Fig). To examine how UPF regulates *ngf-1* mRNA levels, we first showed that UPF did not affect the transcription and synthesis of *ngf-1* mRNA by conducting a RPB-1 ChIP assay (Fig 5C). Since the function of UPF is closely related to mRNA decay, we examined the *ngf-1* mRNA decay rate by addition of thiolutin to block the synthesis of the mRNA, and found that deletion of *upf* resulted in increased stability of *ngf-1* mRNA (Fig 5D). These results indicate that elevated NGF-1 protein was attributed to the impaired decay rate of *ngf-1* mRNA rather than the improvement of transcription activity of *ngf-1* gene under physiological conditions.

**Fig 6:**
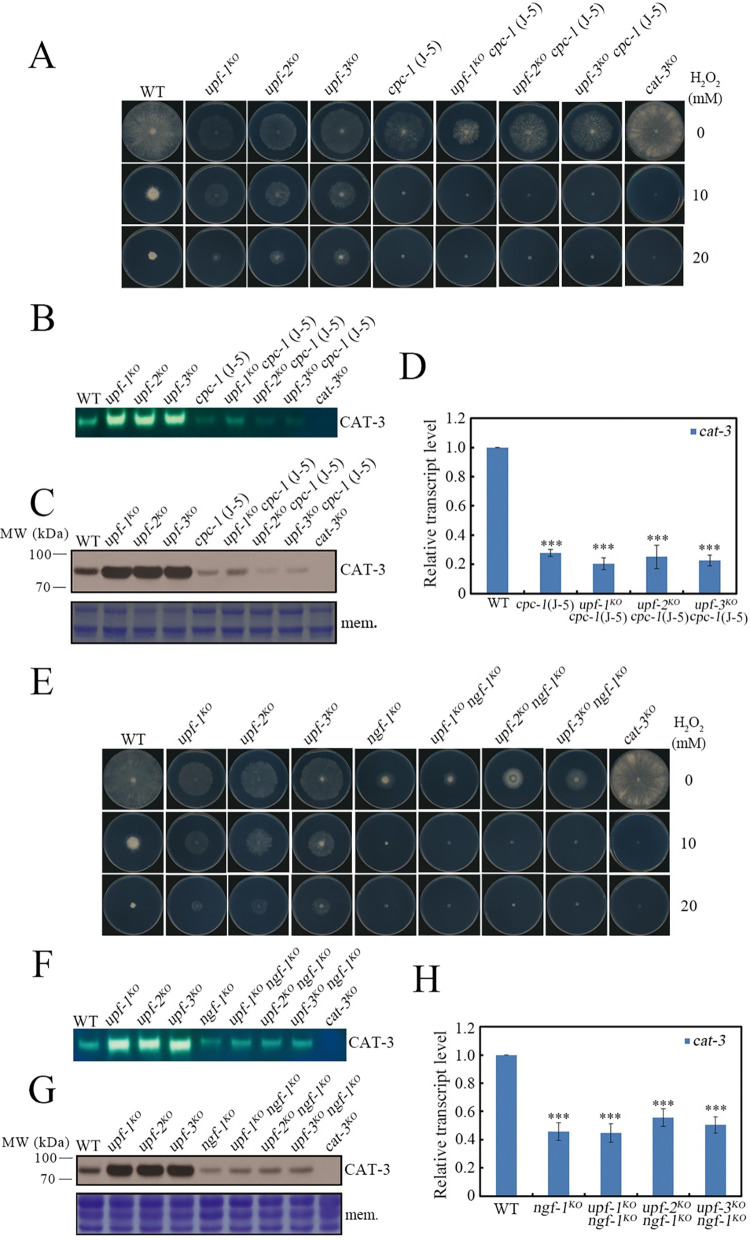
Loss of CPC-1 or NGF-1 effectively reverses the high expression of *cat-3* gene caused by UPF deletion. (A and E) Plate assays analyzing mycelial growth of WT, *upf-1*^*KO*^, *upf-2*^*KO*^, *upf-3*^*KO*^ and *cpc-1* (J-5), *upf-1*^*KO*^ *cpc-1* (J-5), *upf-2*^*KO*^ *cpc-1* (J-5), *upf-3*^*KO*^ *cpc-1* (J-5) strains (A) or *ngf-1*^*KO*^, *upf-1*^*KO*^ *ngf-1*^*KO*^, *upf-2*^*KO*^ *ngf-1*^*KO*^, *upf-3*^*KO*^ *ngf-1*^*KO*^ strains (E) under different H_2_O_2_ concentrations. (B and F) In-gel assays showing the CAT-3 activities of WT, *upf-1*^*KO*^, *upf-2*^*KO*^, *upf-3*^*KO*^ and *cpc-1* (J-5), *upf-1*^*KO*^ *cpc-1* (J-5), *upf-2*^*KO*^ *cpc-1* (J-5), *upf-3*^*KO*^ *cpc-1* (J-5) strains (B) or *ngf-1*^*KO*^, *upf-1*^KO^ *ngf-1*^*KO*^, *upf-2*^*KO*^ *ngf-1*^*KO*^, *upf-3*^*KO*^ *ngf-1*^*KO*^ strains (F). (C and G) Western blot showing the levels of CAT-3 protein in WT, *upf-1*^*KO*^, *upf-2*^*KO*^, *upf-3*^*KO*^ and *cpc-1* (J-5), *upf-1*^*KO*^ *cpc-1* (J-5), *upf-2*^*KO*^ *cpc-1* (J-5), *upf-3*^*KO*^ *cpc-1* (J-5) strains (C) or *ngf-1*^*KO*^, *upf-1*^*KO*^ *ngf-1*^*KO*^, *upf-2*^*KO*^ *ngf-1*^*KO*^, *upf-3*^*KO*^ *ngf-1*^*KO*^ strains (G). The membrane stained by Coomassie blue represented the total protein in each sample and served as the loading control. (D and H) RT-qPCR assays showing the levels of *cat-3* mRNA in WT, *upf-1*^*KO*^, *upf-2*^*KO*^, *upf-3*^*KO*^ and *cpc-1*(J-5), *upf-1*^*KO*^ *cpc-1* (J-5), *upf-2*^*KO*^ *cpc-1* (J-5), *upf-3*^*KO*^ *cpc-1* (J-5) strains (D) or *ngf-1*^*KO*^, *upf-1*^*KO*^ *ngf-1*^*KO*^, *upf-2*^*KO*^ *ngf-1*^*KO*^, *upf-3*^*KO*^ *ngf-1*^*KO*^ strains (H). Error bars indicate S.D. (*n* = 3). *P **<** 0.05; **P **<** 0.01; ***P **<** 0.001. Unpaired Student’s *t* test was used. The *cat-3*^*KO*^ strain was used as the negative control in (A) (B) (C) (E) (F) (G).

To further confirm the regulatory roles of CPC-1 and NGF-1 in *cat-3* gene expression in the *upf* mutants, we constructed *upf cpc-1* (J-5) and *upf ngf-1* double mutants by crossing each *upf* mutant with either *cpc-1* (J-5) or *ngf-1* mutant strain and examined the H_2_O_2_ sensitivities of these double mutants, respectively. Similar to the *cpc-1* (J-5) and *ngf-1* single mutants, these double mutants all exhibited strong H_2_O_2_-sensitive phenotypes (Fig 6A and 6E), indicating that loss of CPC-1 or NGF-1 reversed the H_2_O_2_-resistant phenotypes of the *upf* mutants. Consistent with these phenotypes, the elevated levels of CAT-3 activities (Fig 6B and 6F), CAT-3 protein (Fig 6C and 6G) and *cat-3* mRNA (Fig 6D and 6H) in the *upf* mutants were greatly decreased in the absence of CPC-1 or NGF-1 protein, demonstrating the essential roles of CPC-1 and NGF-1 for the highly expressed *cat-3* gene in the *upf* mutants. Overall, these results suggest that UPF proteins suppress *cat-3* gene expression through mediating *cpc-1* and *ngf-1* mRNA decay under physiological conditions.

### 4. H_2_O_2_ treatment strongly elevates *cat-3* expression to respond to oxidative stress through repressing UPF protein levels in *N*. *crassa*

**Fig 7:**
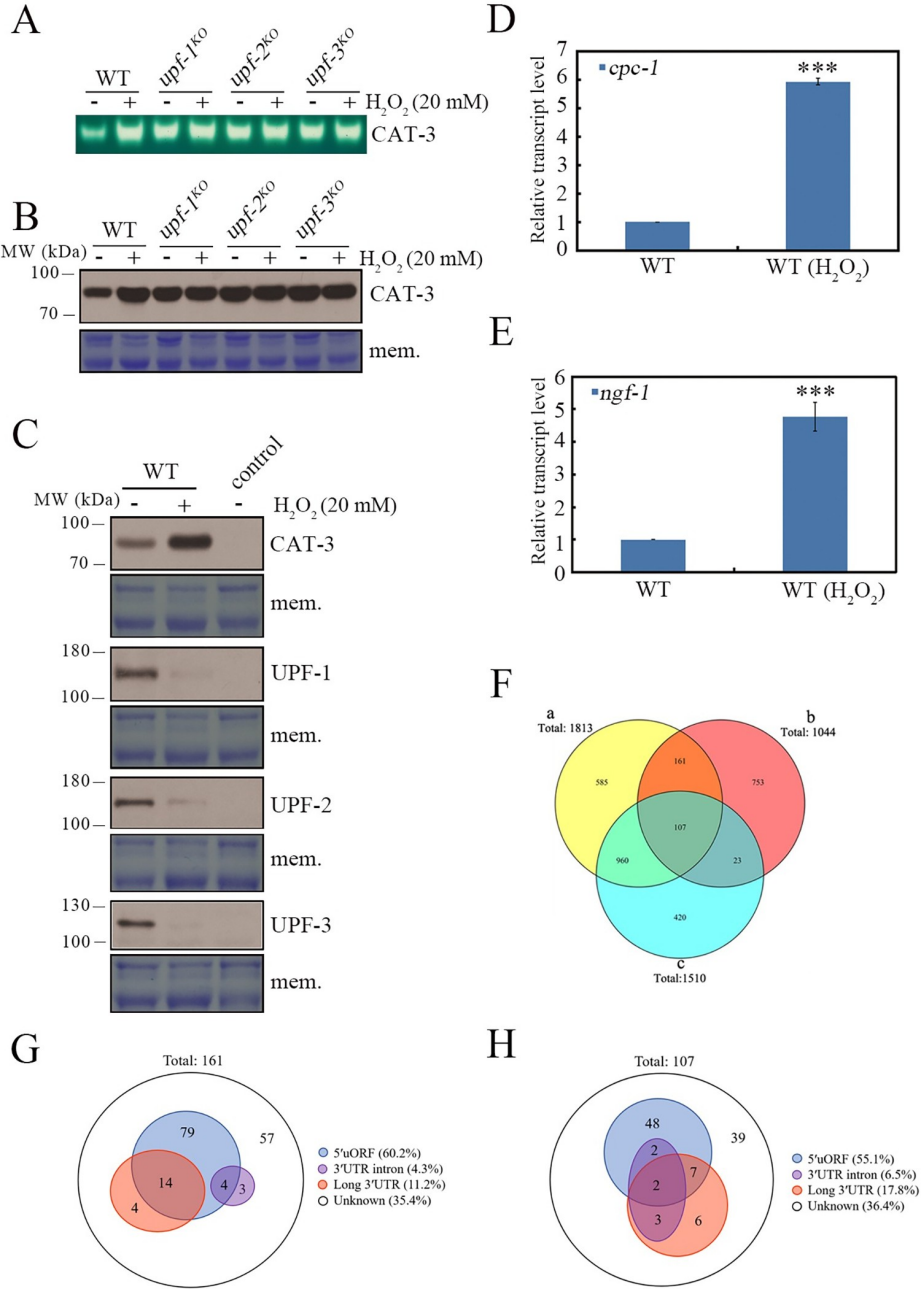
UPF proteins are responsible for dealing with the H_2_O_2_-induced oxidative stress. (A) In-gel assays showing the CAT-3 activities of WT, *upf-1*^*KO*^, *upf-2*^*KO*^ and *upf-3*^*KO*^ strains under different H_2_O_2_ concentrations. (B) Western blot showing the levels of CAT-3 protein in WT, *upf-1*^*KO*^, *upf-2*^*KO*^ and *upf-3*^*KO*^ strains under different H_2_O_2_ concentrations. (C) Western blot showing the levels of CAT-3, UPF-1, UPF-2 and UPF-3 proteins in the WT strain under different H_2_O_2_ concentrations. The corresponding controls indicated *cat-3*^*KO*^, *upf-1*^*KO*^, *upf-2*^*KO*^ and *upf-3*^*KO*^ strains. The membrane stained by Coomassie blue represented the total protein in each sample and served as the loading control. (D and E) RT-qPCR assays showing the levels of *cpc-1* (D) and *ngf-1* (E) mRNA in the WT strain under different H_2_O_2_ concentrations. Error bars indicate S.D. (*n* = 3). *P **<** 0.05; **P **<** 0.01; ***P **<** 0.001. Unpaired Student’s *t* test was used. (F) a. The number of genes upregulated by H_2_O_2_ treatment in the WT strain; b. The number of genes upregulated in the *upf-1*^*KO*^ strain; c. The number of genes upregulated by H_2_O_2_ treatment in the *upf-1*^*KO*^ strain. log2 (Fold change) ≥1, pValue <0.05. (G and H) Distribution of genes with different features upregulated (fold change ≥ 2) in the 161 (G) and 107 (H) genes. The results were obtained from RNA-seq data.

Considering the multilevel effects of UPF proteins on *cat-3* gene expression, we next tested whether UPF proteins are crucial in dealing with H_2_O_2_-induced oxidative stress in *N*. *crassa*. For this purpose, we measured the levels of CAT-3 activities and CAT-3 protein in WT and *upf* strains treated with or without 20 mM H_2_O_2_ in liquid media. As expected, the levels of CAT-3 activities and CAT-3 protein significantly increased in the WT strain upon H_2_O_2_ treatment compared to those without H_2_O_2_ treatment (Fig 7A and 7B). Surprisingly, H_2_O_2_ treatment failed to further elevate their levels in the *upf* mutants (Fig 7A and 7B). These results strongly suggest that the UPF complex plays a critical role in responding to H_2_O_2_-induced oxidative stress in *N*. *crassa*. To determine how UPF-mediated regulation of *cat-3* expression is released during H_2_O_2_-induced oxidative stress, we first examined the expression of *upf* genes upon H_2_O_2_ treatment in WT strain. RT-qPCR analysis revealed that the levels of *upf* mRNAs remained unchanged with or without H_2_O_2_ treatment (S7A–S7C Fig). We next generated specific antibodies of UPF-1, UPF-2, or UPF-3 (S8A–S8C Fig) to examine UPF protein levels. Unexpectedly, western blot analysis showed that H_2_O_2_ treatment significantly decreased the UPF protein levels compared to those without H_2_O_2_ treatment (Fig 7C). Consistent with decreased UPF protein levels, *cpc-1* and *ngf-1* mRNA levels in the WT strain increased upon H_2_O_2_ treatment, similar to those in *upf* mutants (Fig 7D and 7E). These results demonstrate that H_2_O_2_ treatment triggers NMD suppression through downregulating UPF proteins rather than mRNAs, which in turn releases *cat-3* expression to respond to H_2_O_2_-induced oxidative stress.

H_2_O_2_-mediated NMD suppression persuaded us to further analyze the effect of UPF downregulation on genome-wide changes of gene expression profile after H_2_O_2_ treatment. RNA-seq data showed that the expression levels of 1813 genes increased at least twofold after exposure to H_2_O_2_ in the WT strain. Among these 1813 genes, 268 genes (14.8%) were likewise upregulated in the *upf-1*^*KO*^ strain without H_2_O_2_ treatment (Fig 7F). *cat-1*, *cat-2*, and *cat-3* were included in the 268 overlapping genes. Consistent with the elevated mRNA levels of *cat-1* and *cat-2* in *upf-1*^*KO*^ strains without H_2_O_2_ treatment, the activities and protein levels of these two catalases increased in *upf* mutants compared with those in the WT strain (S9A–S9C Fig). Element analysis of these 268 transcripts revealed that 96 genes (35.8%) had zero features of NMD substrates, whereas 172 genes (64.2%) contained at least one feature (5’uORF, 3’UTR intron, or long 3’UTR) of NMD substrates (Fig 7G and 7H). Of these 268 UPF-1-regulated genes, the expression levels of 107 genes (including *cat-1* and *cat-2*) were further increased in the *upf-1*^*KO*^ strains after exposure to H_2_O_2_, whereas 161 genes (including *cat-3*) were not further increased in the *upf-1*^*KO*^ strain during H_2_O_2_ treatment (S3 and S4 Tables). In contrast to the elevated mRNA levels of *cat-1* and *cat-2* in *upf-1*^*KO*^ strain after treatment with H_2_O_2_, catalase activities and protein levels of CAT-1 and CAT-2 decreased in *upf* mutants upon H_2_O_2_ treatment compared to those in the untreated samples (S9A–S9C Fig). GO analysis revealed that these 268 genes were classified as redox-related pathways (Fig 1A). Considering that catalases are heme-proteins, we also analyzed the mRNA levels of enzymes involved in heme synthesis by RNA-seq. Although there was no significant increase for heme synthesis genes expression in the *upf* mutants, the high basal expressions, especially for NCU01546 genes, may be able to maintain a huge reserve pool of heme in the cells (S5 Table). These results suggest that H_2_O_2_-triggered UPF inhibition upregulates redox-related pathways to deal with H_2_O_2_-induced oxidative stress.

### 5. H_2_O_2_-induced degradation of UPF protein is partially dependent on eIF2α phosphorylation

**Fig 8:**
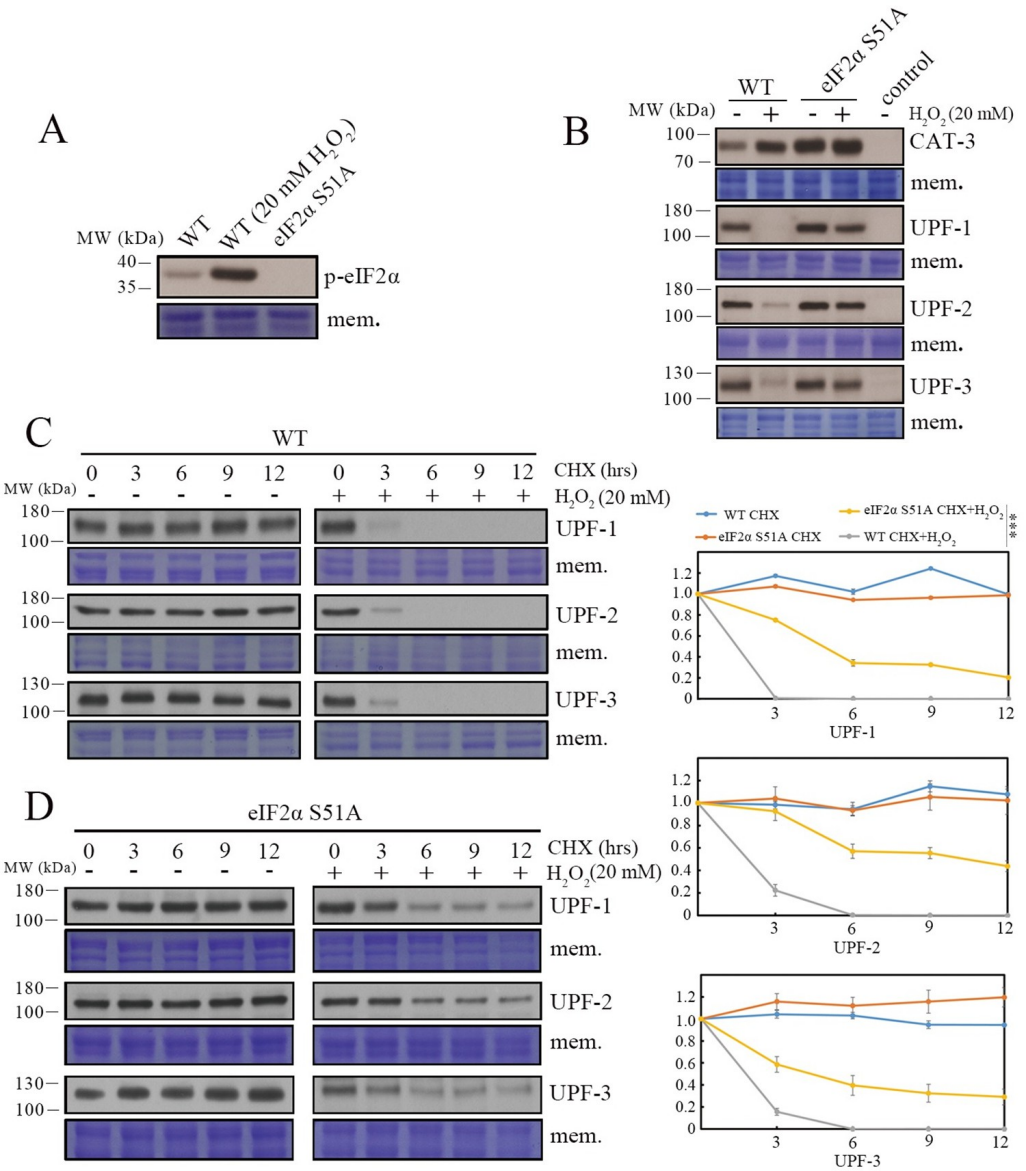
H_2_O_2_-induced degradation of UPF proteins is partially dependent on eIF2α phosphorylation. (A) Western blot showing the levels of eIF2α phosphorylation in the WT strain after the addition of 20 mM H_2_O_2_. The eIF2α S51A strain was used as the negative control. (B) Western blot showing the levels of CAT-3, UPF-1, UPF-2 and UPF-3 proteins in WT and eIF2α S51A strains under 20 mM H_2_O_2_. The corresponding controls indicated *cat-3*^*KO*^, *upf-1*^*KO*^, *upf-2*^*KO*^ and *upf-3*^*KO*^ strains. (C and D) Western blot showing the levels of UPF-1, UPF-2 and UPF-3 proteins in WT (C) and eIF2α S51A (D) strains under the treatment with CHX (cycloheximide) alone or in combination with 20 mM H_2_O_2_, respectively. Quantification of the UPF protein levels was showed on the right. The membrane stained by Coomassie blue represented the total protein in each sample and served as the loading control. Error bars indicate S.D. (*n* = 3). *P **<** 0.05; **P **<** 0.01; ***P **<** 0.001.

Previous studies revealed that eIF2α S51 phosphorylation, the core event of the integrated stress response (ISR), serves as a molecular hub linking external stress response and NMD pathway suppression through globally decreasing protein synthesis [77, 80]. To determine the implications of eIF2α phosphorylation in dealing with H_2_O_2_-induced oxidative stress in *N*. *crassa*, we measured eIF2α S51 phosphorylation levels in the WT strain with or without H_2_O_2_ treatment. As shown in Fig 8A, H_2_O_2_ treatment significantly up-regulated eIF2α S51 phosphorylation levels, indicating that eIF2α S51 phosphorylation likely functions in response to H_2_O_2_-induced oxidative stress in *N*. *crassa*. To further confirm the role of eIF2α S51 phosphorylation in combating oxidative stress, we generated eIF2α S51A and eIF2α S51D mutants, in which the mutation of S51 to A abolishes eIF2α phosphorylation whereas the mutation of S51 to D mimics eIF2α phosphorylation, respectively. As expected, H_2_O_2_ treatment failed to down-regulated UPF protein levels in the eIF2α S51A strain compared to those in the WT and eIF2a S51D strains (Figs 8B and S10A), confirming that eIF2α S51 phosphorylation is critical for oxidative stress-induced NMD suppression by blocking protein synthesis in *N*. *crassa*. We also determined whether H_2_O_2_ treatment affects the degradation of UPF proteins in the WT and eIF2α S51A mutant after the addition of the protein synthesis inhibitor cycloheximide. Surprisingly, H_2_O_2_-induced oxidative stress strongly increased degradation of UPF proteins in the WT strain. However, eIF2α S51A mutation significantly restored the stability of UPF proteins even under H_2_O_2_ treatment (Fig 8C and 8D), suggesting that the eIF2α phosphorylation likely promotes the degradation of UPF proteins through an unknown mechanism. CPC-3 was reported as the main kinase responsible for eIF2α phosphorylation in *N*. *crassa* [81, 82]. However, loss of CPC-3 failed to block the down-regulation of UPF protein levels and up-regulation of CAT-3 protein levels under H_2_O_2_ treatment (S10B Fig), implying that there are other kinases that respond to H_2_O_2_-induced oxidative stress in *N*. *crassa* [83–85].

## Discussion

**Fig 9:**
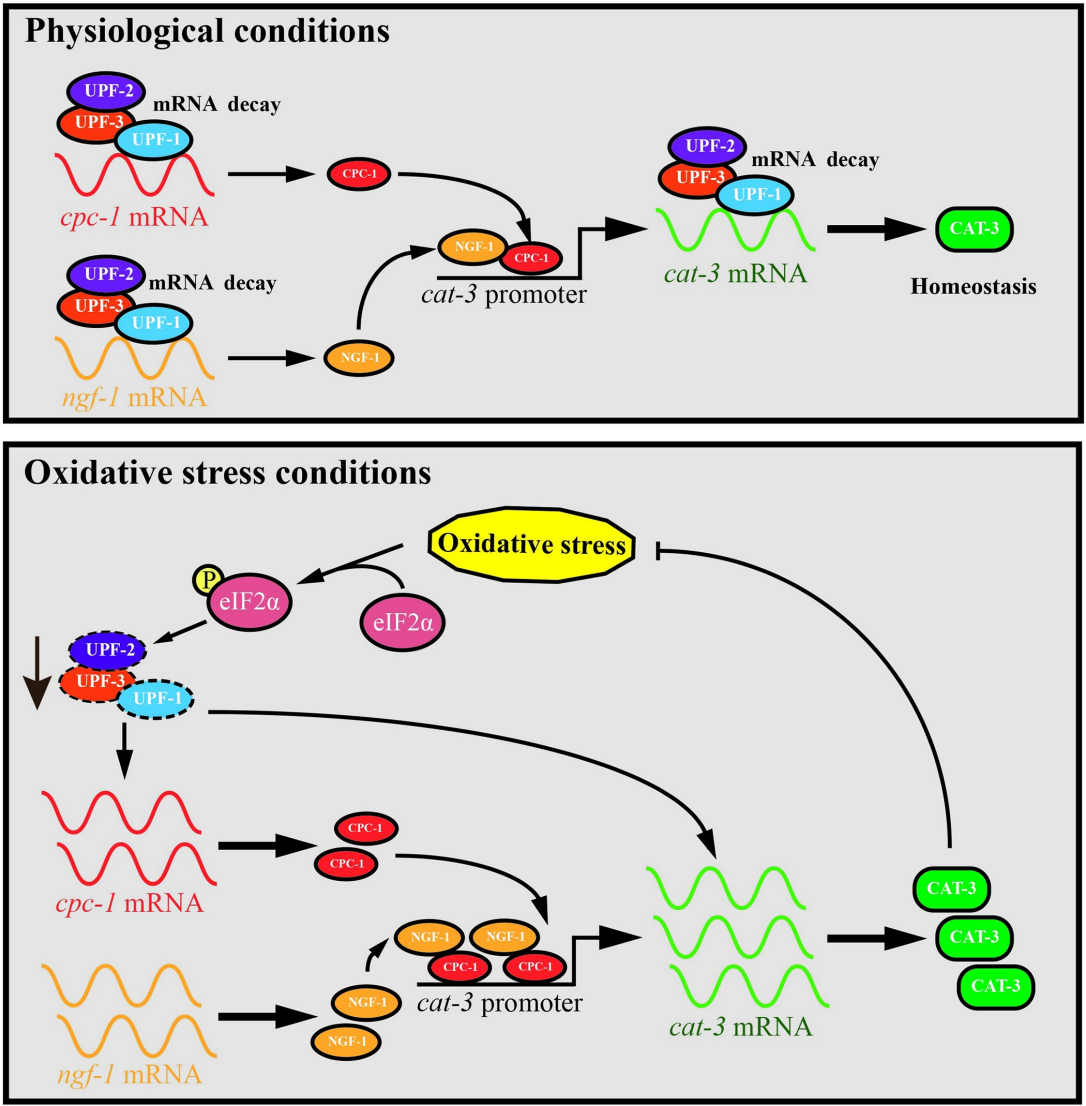
The working model depicting the UPF proteins-mediated multilevel regulation of *cat-3* gene expression in *N*. *crassa*. Under physiological conditions, UPF proteins not only directly promote the degradation of *cat-3* mRNA, but indirectly repress *cat-3* gene transcription by boosting *cpc-1* and *ngf-1* mRNAs decay, which encode the critical positive regulators for *cat-3* gene expression, eventually maintaining the homeostasis of CAT-3 protein and activity. Once cells are subjected to oxidative stress, UPF proteins are degraded partially through an eIF2α phosphorylation-dependent manner, thus the stabilities of *cpc-1*, *ngf-1* and *cat-3* mRNAs are significantly improved, ultimately leading to a significant increase of CAT-3 activity to deal with the excessive oxidative stress.

Recent studies have clearly demonstrated that the NMD pathway contributes to appropriate cellular responses to various environmental stresses by dynamically adjusting the mRNAs levels of key inducible genes. Our data further complement these previous reports by showing how NMD factors regulate *cat-3* expression during H_2_O_2_-induced oxidative stress. Under physiological conditions, UPF proteins not only control the stability of *cat-3* transcripts, but also indirectly repress *cat-3* gene transcription by boosting *cpc-1* and *ngf-1* mRNA decay to maintain ROS homeostasis. Moreover, we identified that UPF proteins serve as a sensitive factor for H_2_O_2_-induced oxidative stress in *N*. *crassa*. When subjected to H_2_O_2_ treatment, UPF proteins are degraded, which in turn triggers activation of *cat-3* expression to eliminate oxidative stress in cells (Fig 9). Our results therefore demonstrate the multilevel regulatory roles of *N*. *crassa* UPF complex in *cat-3* gene expression under physiological conditions as well as oxidative stress conditions.

### The functions of NMD under physiological conditions

As a robust inducible gene, the expression pattern of *cat-3* fluctuates with changes in ROS levels during the growth and development of *N*. *crassa*, which is mainly achieved by competition between positive and negative regulators. Our previous study showed that the transcription factor CPC-1 and histone acetyltransferase NGF-1 are responsible for activating *cat-3* transcription and resisting H_2_O_2_-induced oxidative stress [59]. Here, we demonstrated that UPF proteins, the key negative regulators, not only directly promote *cat-3* mRNA decay, but indirectly repress *cat-3* gene transcription by boosting *cpc-1* and *ngf-1* mRNAs decay, eventually maintaining an optimal amount of CAT-3 protein to deal with the fluctuation of ROS under physiological conditions. Consistent with our study, NMD-mediated decay of *Atf4* mRNA, the functional homologue of *N*. *crassa cpc-1* gene in mammals, also controls the expression patterns of key response genes for other stress, such as hypoxia, amino acid deprivation and ER stress [86, 87]. Previous study revealed that mouse *Atf4* possesses two upstream open reading frames (uORF1 and uORF2) that are potential features for triggering NMD-mediated decay of normal transcripts. The translation of the second uORF is favoured under normal conditions, which leads to NMD activation and low levels of ATF4 protein [33]. Four uORFs are required for translational control of yeast GCN4. The ribosomes start scanning at uORF1 and re-initiate at downstream uORFs that blocks the translation of GCN4 coding sequence [77]. In the *N*. *crassa*, *cpc-1* gene also contains two uORFs, implying that such regulatory mechanisms may apply to *cpc-1* gene [31, 74, 76]. Importantly, about 22–50% of eukaryotic mRNAs contain putative uORFs with possible regulatory effects according to their propensities to trigger NMD, thus, their translations are closely linked to their estimated sensitivities to the NMD machinery [1, 8, 88, 89]. Unlike *cpc-1 gene*, *ngf-1* gene harbors a long 3’UTR that is another potential feature for NMD-mediated decay of normal transcripts. Considering the regulatory functions of NGF-1 in various biological events, NMD-mediated balance of *ngf-1* expression level seems more complicated in addition to coping with oxidative stress [60, 61, 90]. Considering the diversity of substrates targeted by the NMD pathway, we cannot rule out the possibility that other substrates may also contribute to UPF’s repressive role in *cat-3* homeostasis in addition to *cpc-1* and *ngf-1* transcripts. Intriguingly, the NMD pathway probably possesses a conserved feedback regulatory network, because most eukaryotic transcripts of NMD factors contain at least one feature, uORFs or the long 3’UTR, for triggering NMD autoregulation [8, 62, 72, 91]. For example, *upf-1* mRNA contains two uORFs and a long 3’UTR and becomes more stable in *upf-2*^*KO*^ strain [8]. This autoregulation provides a potential buffering mechanism to avoid excessive activation of NMD factors which may cause dysfunction of a bulk of NMD substrates in cells during growth and development.

*N*. *crassa* employs such an elaborate mechanism to actively maintain low levels of CAT-3 under physiological conditions. The system is able to react quickly to sudden increases in external H_2_O_2_, which could mean a life-threatening situation. Releasing the negative control of the NMD would ensure a rapid response with a burst of extracellular CAT-3. Once the stress is overcome, a much lower and regular amounts of CAT-3 would be secreted to maintain the cell wall integrity with its catalase and chaperone activities [55, 92]. By maintaining low levels of CAT-3, *N*. *crassa* ensures a basal level of ROS that can be utilized for regulating the complex developmental processes, such as asexual and sexual reproduction, hyphal growth, and differentiation specialized structures [93–96]. For example, a hyperoxidant state has been reported to occur at the beginning phase of some morphogenetic transitions such as hyphal adhesion, aerial mycelium formation, and conidium formation in *N*. *crassa* [55, 97–100]. In addition, catalase overexpression increases the pathogenicity and reduces the germination time of other fungi [101]. Last, overexpression of human mitochondrial catalase leads to elevated inflammatory signaling by activating the redox-sensitive NF-κB in mice macrophages [102, 103]. Therefore, the precise regulation of intracellular catalase expression seems highly desirable under physiological conditions.

### The functions of NMD in adaptation to stress and environment

In eukaryotes, various stresses including amino acid deprivation, viral infection and ER stress can activate a common adaptive pathway, termed integrated stress response (ISR). A correlation was made between the induction of *N*. *crassa* catalase genes by H_2_O_2_-induced oxidative stress and those enzymes for resistance to the same stress. During ISR, eukaryotic translation initiation factor 2α (eIF2α) is phosphorylated by GCN2, PKR, HRI or PER kinases, eventually leading to a global attenuation of cap-dependent translation and the concomitant induction of ISR-specific mRNAs to re-establish cellular homeostasis [77, 104]. Translations of these ISR-specific mRNAs may not require cap recognition by the eIF4F complex, but rely on a re-initiation mechanism or the direct recruitment of ribosomes to internal ribosome entry sites (IRES) [77, 105]. The activity of the NMD pathway is critical to silence ISR under physiological condition until enough stress promotes NMD suppression because many ISR-specific transcripts are natural NMD targets, such as *Atf4*. We have previously revealed that the histone acetyltransferase NGF-1 and the transcription factor CPC-1 are required to maintain *cat-3* expression. Our research further showed that H_2_O_2_-induced oxidative stress significantly down-regulates the activity of the NMD pathway, which in turn rescues *cpc-1* and *ngf-1* mRNA levels to enhance fitness and survival through elevating *cat-3* expression. Unlike the regulatory mechanisms in *N*. *crassa*, *S*. *pombe* Upf1 and Csx1, an RNA-binding protein, work in the same pathway to stabilize *atf1* and *ctt1* mRNAs during oxidative stress and thus *S*. *pombe upf* mutants are sensitive to oxidative stress [38].

It remains a key question as to how the oxidative stress triggers NMD suppression in the *N*. *crassa*. Several lines of evidence suggest that cellular stress achieves this by triggering eIF2α phosphorylation. eIF2α phosphorylation leads to the formation of stress granules (SGs) that sequester several key NMD factors in translationally arrested mRNAs and thus deplete NMD factors in the surrounding cytosol [106, 107]. Therefore, NMD factors would not have access to translationally active mRNAs in the cytosol in stressed cells. In this study, we found that H_2_O_2_-induced oxidative stress triggers NMD suppression through decreasing protein levels of UPFs but not the levels of their mRNA. Knockdown of SMG1, a key kinase in the NMD pathway, decreases the number of SGs when treated with sodium arsenite [108], implying that a possible feedback loop maintains RNA metabolism homeostasis by buffering both RNA decay and translation in response to stress. Similarly, in our study, eIF2α S51A mutation but not eIF2α S51D failed to inhibit the synthesis of UPF proteins under H_2_O_2_ treatment. Interestingly, eIF2α S51A mutation also significantly restored the stability of UPF proteins even under H_2_O_2_ treatment in WT strain. These results suggest that oxidative stress can blunt the NMD pathway by eIF2α phosphorylation. We found that with the loss of CPC-3, the homolog of mammalian eIF2α kinases in *N*. *crassa*, UPF proteins synthesis is still inhibited under H_2_O_2_ treatment, suggesting that there are other eIF2α kinases that respond to H_2_O_2_-induced oxidative stress in *N*. *crassa*. Tuning NMD activity is also important for tumor cells to adapt to the tumor microenvironment. NMD is decreased during tumor formation in order to overcome microenvironment-related stresses, such as hypoxia and ER stress. Oxidative stress has been shown to trigger ER stress pathway, activate apoptosis pathways and cause cell death in liver tissue [109]. Therefore, activation of eIF2α kinases by oxidative stress probably occurs through a crosstalk with other stresses. Upon H_2_O_2_ stress, the membrane receptors can sense and transfer oxidative stress signals via calcium or protein kinase pathways to UPF proteins, then CPC-1 and NGF-1, resulting in elevated *cat-3* transcription. CAT-3 has an N-terminal signal peptide that is processed [56] and possibly used for enzyme secretion to protect *N*. *crassa* against exogenous oxidative stress [55, 110]. Besides extracellular oxidative stress, intracellular ROS burst induced by curcumin has been reported to inhibit the NMD pathway [111, 112]. Therefore, other conditions that increase intracellular H_2_O_2_, such as cytochrome *c* peroxidase deficiency, probably also trigger *cat-3* transcription through inhibiting NMD mechanisms. Interestingly, in addition to catalases, ROS scavenger molecule glutathione has also been reported to be exploited to cope with MYC-induced intracellular toxic ROS by activating the ISR and suppressing NMD [113], further expanding the scope of NMD substrates for intracellular ROS regulation. In summary, our study reveals that UPF factors play an important role in responding to ROS stress by activating *cat-3* transcription which cleans up excess ROS.

## Materials and methods

### Strains and culture conditions

In this study, the *N*. *crassa* 87–3 (*bd*, *a*) strain with *bd* mutation (*ras-1*^*T79I*^) and FGSC 4200 (ORS-SL6a) strain were used as the wild-type strains [114]. The *ku70*^*RIP*^ (*bd*, *a*) strain in which the *ku70* ORF was mutated with multiple premature stop codons by repeat-induced point mutation [115] was used as the host strain for creating different *Hygromycin B* (*hph*) knockout strains (*upf-1*^*KO*^, *upf-2*^*KO*^ and *upf-3*^*KO*^) because loss-of-function of Ku70 protein causes extraordinarily high homologous recombination efficiency and low non-homologous end-joining rate [116]. The *cat-3*^*KO*^, *cpc-1* (j-5) and *ngf-1*^*KO*^ strains generated previously were also used in this study [59, 117]. The newly double mutants were generated by crossing, *upf-1*^*KO*^ *upf-2*^*KO*^, *upf-1*^*KO*^ *upf-3*^*KO*^, *upf-2*^*KO*^ *upf-3*^*KO*^, *upf-1*^*KO*^ *cpc-1* (j-5), *upf-2*^*KO*^ *cpc-1* (j-5), *upf-3*^*KO*^ *cpc-1* (j-5), *upf-1*^*KO*^ *ngf-1*^*KO*^, *upf-2*^*KO*^ *ngf-1*^*KO*^ and *upf-3*^*KO*^ *ngf-1*^*KO*^. The *upf-1*^*KO*^, pqa-Myc-UPF-1, *upf-1*^*KO*^, pqa-Myc-UPF-1^△CHD^, *upf-1*^*KO*^, pqa-Myc-UPF-1^△ATPBD^, *upf-1*^*KO*^, pqa-Myc-UPF-1^△RBD^ and *upf-1*^*KO*^, pqa-Myc-UPF-1^△SQD^ transformants were generated by transferring each construct into the *his-3* locus of *upf-1*^*KO*^ (*his-3*, *a*) host strain. Likewise, the *upf-2*^*KO*^, pqa-Myc-UPF-2, *upf-2*^*KO*^, pqa-Myc-UPF-2^△MIF4G-1^, *upf-2*^*KO*^, pqa-Myc-UPF-2^△MIF4G-2^, *upf-2*^*KO*^, pqa-Myc-UPF-2^△MIF4G-3^, *upf-2*^*KO*^, pqa-Myc-UPF-2^△UPF1BD^ and *upf-3*^*KO*^, pqa-Myc-UPF-3 transformants were created. All strains used here possess the same *bd* background. The *Neurospora crassa* locus identifier (NCU) and Fungal Genetics Stock Center (FGSC) numbers of the *N*. *crassa upf-1*, *upf-2*, *upf-3*, *cat-3* and *ngf-1* genes are NCU04242 (FGSC11229/FGSC11230), NCU05267 (FGSC#15705/FGSC#15706), NCU03435 (FGSC#11679/FGSC#11680), NCU00355 (FGSC#11201/FGSC#11202) and NCU10847 (FGSC#16229). The *cpc-1* (j-5) band mutant generated from the crossing of *cpc-1* (j-5) (FGSC#4433 and #4434) with 87–3 (*bd*, *a*) strain [59]. Strains list see in S6 Table.

Conidia of indicated strain were inoculated in Petri dishes with 50 mL minimal medium (2% glucose) and cultured at 25°C in constant light until the exponential growth phase of mycelia. The mycelial mat was cut with a specific puncher for quantification. Then, small mycelial disks were transferred to Erlenmeyer flasks with 50 mL minimal medium and grown at 25°C with shaking for 18 hours under constant light. When quinic acid (QA) was added, mycelial disks were grown in 50 mL low-glucose medium (1 **×** Vogel’s, 0.1% glucose and 0.17% arginine) with 10^**−**2^ M QA.

### Generation of antisera against NGF-1, UPF-1, UPF-2, and UPF-3

The GST-NGF-1 (amino acids Ala34-Glu280), GST-UPF-1 (amino acids His690-Gln892), GST-UPF-2 (amino acids Pro202-Met488) and GST-UPF-3 (amino acids Thr236-Thr548) fusion proteins were expressed in *Escherichia coli* BL21 cells, respectively. Purified recombinant proteins were used as the antigens to generate rabbit polyclonal antiserums as previously described [116]. The CPC-1, RPB-1, and CAT-3 antibodies generated previously were also used in this study [59, 117, 118].

### Plate assay

The medium for plate assays contained 1 × Vogel’s, 3% sucrose, 1.5% (w/v) agar with different concentrations of H_2_O_2_ and QA. About 1 week old conidia of specific strains were inoculated in Petri dishes with 50 mL liquid medium containing 1× Vogel’s salts and 2% glucose under static culture condition at 25°C for 48 hours. Disks of mycelium film were cut with an iron borer and placed in Petri dishes containing medium. WT or mutant strains were inoculated at the center of the disks and grown under constant light at 25°C on the medium containing 0, 10, or 20 mM H_2_O_2_. When the WT strain almost completely covered the medium without H_2_O_2_, all the plates were scanned, and the average growth rate of each strain relative to that in medium without H_2_O_2_ (0 mM) was calculated. The growth diameter of WT and *upf* mutants under 0 mM H_2_O_2_ was defined as their respective reference unit "1", and the relative growth of WT and *upf* mutants under 10 mM and 20 mM H_2_O_2_ was calculated, respectively. Each experiment was performed at least three times independently [73].

### In-gel assay for catalases activity

Cell extracts from the well-cultured mycelium disks were used for performing in-gel assay. Protein extraction and quantification prepared for the in-gel assay were same as previously described [117, 119, 120]. Equal amounts of total protein (40 μg) were loaded into each protein lane of 7.5% native poly-acrylamide slab gel. After electrophoresis, the gel was rinsed with ddH_2_O and subsequently soaked in 10 mM H_2_O_2_ with gently shaking for 10 min. Then, the gel was straightway transferred into a mixture of freshly prepared 1% potassium hexacyanoferrate (III) and 1% iron (III) chloride hexahydrate. Catalase activity was visualized as a band where H_2_O_2_ was decomposed by catalases.

### The stained colonies for testing extracellular catalase

Little amount of a-week conidia were resuspended in 1 mL ddH_2_O, and 0.5 μL of those were incubated on solid medium containing 1 × Vogel’s, 1.5% agar, 1 × Fig’s and 1.5% sorbose at 30°C for 3–4 days. The solid media with colonies in Petri dishes were treated with 7 mM H_2_O_2_ for 15 min, and immersed in a mixture of freshly prepared 1% potassium hexacyanoferrate (III) and 1% iron (III) chloride hexahydrate for 1 min. Extracellular catalases were visualized as a transparent circle (halo) where H_2_O_2_ was decomposed by catalases.

### Protein and RNA analyses

Cell extracts from the well-cultured mycelium disks were used for performing protein and RNA analyses. For protein analyses, protein extraction, quantification, western blot assay and protein degradation assay were performed as described previously [121, 122]. Equal amounts of total protein (40 μg) were loaded into each protein lane. After electrophoresis, proteins were transferred onto PVDF membrane by electroblot. Western blot assay was performed by using antibodies against the proteins of interest. After Western blot analyses, the membranes stained by Coomassie blue (0.25% Coomassie brilliant blue R250 (CAS#6104-59-2), 45% methanol, 10% glacial acetic acid) for 10 minutes followed by decolorizing with decolorization solution (45% methanol, 10% glacial acetic acid) for 20 minutes. For RNA analyses, total RNA was extracted with TRIzol agent and treated with DNaseI to digest genomic DNA. Each RNA sample (5 mg) was subjected to reverse transcription with Maxima H Minus reverse transcriptase (Thermo Fisher Scientific #M1682) and then amplified by real-time qPCR (7500; ABI) with AceQ Universal SYBR qPCR Master Mix (Q511; Vazyme) and primer pairs (S7 Table). The results were normalized by the expression level of tubulin gene. Transcription inhibitor thiolutin was used for RNA decay assay. The relative value of gene expression was calculated using the 2^-△△CT^ method by comparing the cycle number for each sample to that for the untreated control [123]. The results were normalized by the expression level of 18s RNA gene.

### ChIP-qPCR

Chromatin immunoprecipitation (ChIP) assays were performed as previously described [124]. Briefly, tissues were fixed with 1% formaldehyde for 15 min, and then treated with 125 mM glycine for 5 min to stop the cross-linking reaction. After harvest, cross-linked tissues were ground (0.5 g) and re-suspended in 6 mL lysis buffer containing protease inhibitors. Chromatin was sheared by sonication to ∼500 bp fragments. 1 mL (2 mg/mL) protein was used as per immunoprecipitation and 10 μL was kept as the input DNA. ChIP was performed with 5 μL antibody to RPB-1 and 5 μL antibody to CPC-1 [59, 118]. Immunoprecipitated DNA was enriched with the GammaBind G Sepharose beads (17-0885-02; GE Healthcare) and eluted using elution buffer (1% SDS and 0.1 M NaHCO_3_). Finally, purified DNA was quantified by real-time qPCR (7500; ABI) with AceQ Universal SYBR qPCR Master Mix (Q511; Vazyme) and primer pairs (S8 Table). Occupancies were normalized by the input DNA and presented as a percentage of input DNA.

### RNA seq and analysis

The *upf-1*^*KO*^ and WT strains were grown and the RNA samples were from independent triplicate pools, respectively. The NovaSeq 6000 system used to perform RNA-seq analysis. The sequencing was performed on an Illumina HiSeq 2000 at Anuo Youda Gene Technology (Beijing) Co., LTD, China. Feature Counts [125] was used to count the reads. After differentially expressed genes were obtained by DESeq2 (Version:1.38.2), we selected significantly upregulated genes for GO analysis (log2FoldChange> = 1, pValue<0.05) by STRING website. The scatter plot and bar plot were drawn using ggplot2 (version: 4.2.4). Meanwhile, VennDiagram (version:1.7.3) was used to map the significantly upregulated genes. Hisat2 [126] was used as the aligner to map the reads to the reference genome [*N*. *crassa* OR74A (NC12)].

## Supporting information

S1 Fig The activities and protein levels of CAT-3 were greatly increased in *upf*^*KO*^ (*nbd*) strains. (A) Growth phenotypes of WT, *upf-1*^*KO*^, *upf-2*^*KO*^ and *upf-3*^*KO*^ strains on slants. (B) Extracellular catalases assay showing the extracellular levels of CAT-3 in the WT and *upf* mutants. Extracellular catalases were visualized as a transparent circle (halo) where H_2_O_2_ was decomposed by catalases. WT clones were stained on day 3, and *upf* mutants clones were stained on day 4 when they reached the same size as WT clones. (C) In-gel assays showing the CAT-3 activities of WT (FGSC 4200, *nbd*), *upf-1*^*KO*^ (*nbd*), *upf-2*^*KO*^ (*nbd*) and *upf-3*^*KO*^ (*nbd*) strains. (D) Western blot showing the levels of CAT-3 protein in WT (FGSC 4200, *nbd*), *upf-1*^*KO*^ (*nbd*), *upf-2*^*KO*^ (*nbd*) and *upf-3*^*KO*^ (*nbd*) strains. The membrane stained by Coomassie blue represented the total proteins in each sample and served as the loading control.
(TIF)

S2 Fig UPF proteins function in the same pathway to repress *cat-3* gene expression. (A) Plate assays analyzing mycelial growth (left) and relative growth statistics (right) of the WT, *upf-1*^*KO*^, *upf-2*^*KO*^, *upf-3*^*KO*^ strains and *upf-1*^*KO*^ *upf-2*^*KO*^, *upf-2*^*KO*^ *upf-3*^*KO*^, *upf-1*^*KO*^ *upf-3*^*KO*^ double mutants under different H_2_O_2_ concentrations. (B) In-gel assays showing the CAT-3 activities of WT, *upf-1*^*KO*^, *upf-2*^*KO*^, *upf-3*^*KO*^ strains and *upf-1*^*KO*^ *upf-2*^*KO*^, *upf-2*^*KO*^ *upf-3*^*KO*^, *upf-1*^*KO*^ *upf-3*^*KO*^ double mutants. (C) Western blot showing the levels of CAT-3 protein in WT, *upf-1*^*KO*^, *upf-2*^*KO*^, *upf-3*^*KO*^ strains and *upf-1*^*KO*^ *upf-2*^*KO*^, *upf-2*^*KO*^ *upf-3*^*KO*^, *upf-1*^*KO*^ *upf-3*^*KO*^ double mutants. The membrane stained by Coomassie blue represented the total protein in each sample and served as the loading control. Error bars indicate S.D. (*n* = 3). *P ***<*** 0.05; **P ***<*** 0.01; ***P ***<*** 0.001. The *cat-3*^*KO*^ strain was used as the negative control in (A) (B) (C).
(TIF)

S3 Fig UPF-1 protein is highly conserved in eukaryotes and the conserved domains of UPF-1 is required for the H_2_O_2_ resistance phenotype of *upf-1*^*KO*^ strain. (A) Amino acid sequence alignment of *Neurospora crassa* UPF-1 protein with its homologous proteins in *Schizosaccharomyces pombe*, *Saccharomyces cerevisiae*, *Drosophila melanogaster*, *Mus musculus* and *Homo sapiens*. (B) Western blot showing the protein levels of Myc-UPF-1 in the different deletion strains across UPF-1 coding region at exogenous locus. The arrow shows the specific band. (C) Plate assays analyzing mycelial growth of the different deletion strains across UPF1 coding domain at exogenous locus driven by *qa-2* promoter under 0, 10, 20 mM H_2_O_2_. The *cat-3*^*KO*^ strain was used as the negative control. Quinic acid (QA) was used to induce the *qa-2* promoter.
(TIF)

S4 Fig UPF-2 protein is relatively conserved in eukaryotes and the conserved domains of UPF-2 is required for the H_2_O_2_ resistance phenotype of *upf-2*^*KO*^ strain. (A) Amino acid sequence alignment of *Neurospora crassa* UPF-2 protein with its homologous proteins in *Schizosaccharomyces pombe*, *Saccharomyces cerevisiae*, *Drosophila melanogaster*, *Mus musculus* and *Homo sapiens*. (B) Western blot showing the protein levels of Myc-UPF-2 in the different deletion strains across UPF-2 coding region at exogenous locus. (C) Plate assays analyzing mycelial growth of the different deletion strains across UPF-2 coding domain at exogenous locus driven by *qa-2* promoter under 0, 10, 20 mM H_2_O_2_. The *cat-3*^*KO*^ strain was used as the negative control. Quinic acid (QA) was used to induce the *qa-2* promoter.
(TIF)

S5 Fig Deletion of UPF-1 has no effect on CPC-1 protein stability. (A) Putative NMD-inducing features (uORFs) in *cat-3* transcript. (B) RT-qPCR assays showing the relative degradation ratio of *Nc2β* mRNA in WT, *upf-1*^*KO*^, *upf-2*^*KO*^ and *upf-3*^*KO*^ strains after the addition of thiolutin. Error bars indicate S.D. (*n* = 3). *P **<** 0.05; **P **<** 0.01; ***P **<** 0.001. Unpaired Student’s *t* test was used. (C) Putative NMD-inducing features (uORFs) in *cpc-1* transcript. (D) Western blot showing the degradation of CPC-1 protein in WT and *upf-1*^*KO*^ strains after the addition of cycloheximide (CHX). Quantification of the CPC-1 protein level was showed below. The membrane stained by Coomassie blue represented the total protein in each sample and served as the loading control.
(TIF)

S6 Fig Deletion of UPF-1 has no effect on NGF-1 protein stability. (A) Putative NMD-inducing features (long 3’UTR) in *ngf-1* transcript. (B) Immunodetection of NGF-1 in the WT strain using polyclonal antiserum that specifically recognizes endogenous NGF-1 protein. The *ngf-1*^*KO*^ strain was used as the negative control. (C) Western blot showing the degradation of NGF-1 protein in WT and *upf-1*^*KO*^ strains after the addition of cycloheximide (CHX). Quantification of the NGF-1 protein level was showed below. The membrane stained by Coomassie blue represented the total protein in each sample and served as the loading control.
(TIF)

S7 Fig H_2_O_2_ treatment has no effect on *upf* mRNAs levels in the WT strain. RT-qPCR assays showing the levels of *upf-1* (A), *upf-2* (B) and *upf-3* (C) mRNAs in the WT strain with or without H_2_O_2_ treatment. Error bars indicate S.D. (*n* = 3). N.S. no significance. Unpaired Student’s *t* test was used.
(TIF)

S8 Fig Polyclonal antiserums could specifically recognize endogenous UPF-1, UPF-2 and UPF-3 proteins, respectively. Immunodetection of UPF-1 (A), UPF-2 (B) and UPF-3 (C) in the WT strain using polyclonal antiserum that specifically recognizes endogenous UPF proteins. *upf-1*^*KO*^, *upf-2*^*KO*^ and *upf-3*^*KO*^ strains were used as the negative control in (A) (B) (C), respectively.
(TIF)

S9 Fig The catalase activities and protein levels of CAT-1 and CAT-2 decreased in the *upf* mutants after H_2_O_2_ treatment. (A) In-gel assays showing the CAT-1 and CAT-2 activities of WT, *upf-1*^*KO*^, *upf-2*^*KO*^ and *upf-3*^*KO*^ strains with or without H_2_O_2_ treatment. (B) and (C) Western blot showing the protein levels of CAT-1 and CAT-2 in WT, *upf-1*^*KO*^, *upf-2*^*KO*^ and *upf-3*^*KO*^ strains with or without H_2_O_2_ treatment. The membrane stained by Coomassie blue represented the total proteins in each sample and served as the loading control.
(TIF)

S10 Fig H_2_O_2_ treatment induces the degradation of UPF proteins in eIF2α S51D and *cpc-3*^*KO*^ strains. (A) Western blot showing the levels of CAT-3, UPF-1, UPF-2 and UPF-3 proteins in WT, eIF2α S51A and eIF2α S51D strains under 20 mM H_2_O_2_. (B) Western blot showing the protein levels of CAT-3, UPF-1, UPF-2 and UPF-3 in WT and *cpc-3*^*KO*^ strains under 20 mM H_2_O_2_. The corresponding controls indicated *cat-3*^*KO*^, *upf-1*^*KO*^, *upf-2*^*KO*^ and *upf-3*^*KO*^ strains. The membrane stained by Coomassie blue represented the total protein in each sample and served as the loading control.
(TIF)

S1 Table GO Category analysis in the *upf-1*^*KO*^ strain. (XLSX)

S2 Table A total of genes were greatly upregulated in the *upf-1*^*KO*^ strain. (XLSX)

S3 Table A total of 107 genes were further upregulated in the *upf-1*^*KO*^ strain by H_2_O_2_ treatment. (XLSX)

S4 Table A total of 161 genes were not further upregulated in the *upf-1*^*KO*^ strain by H_2_O_2_ treatment. (XLSX)

S5 Table Analysis of the basal and fold change of heme synthesis genes expression in the WT and *upf-1*^*KO*^ strains. (XLSX)

S6 Table Strains used in this study. (XLSX)

S7 Table The primers for RT-qPCR assays. (XLSX)

S8 Table The primers for ChIP-qPCR assays. (XLSX)
