# Supplementary figures and images for: Sensing of H2O2-induced oxidative stress by the UPF factor complex is crucial for activation of catalase-3 expression in Neurospora
Source: PLoS Genet. 2023 Oct 16;19(10):e1010985. doi: 10.1371/journal.pgen.1010985 (PMC10578600; doi:10.1371/journal.pgen.1010985)

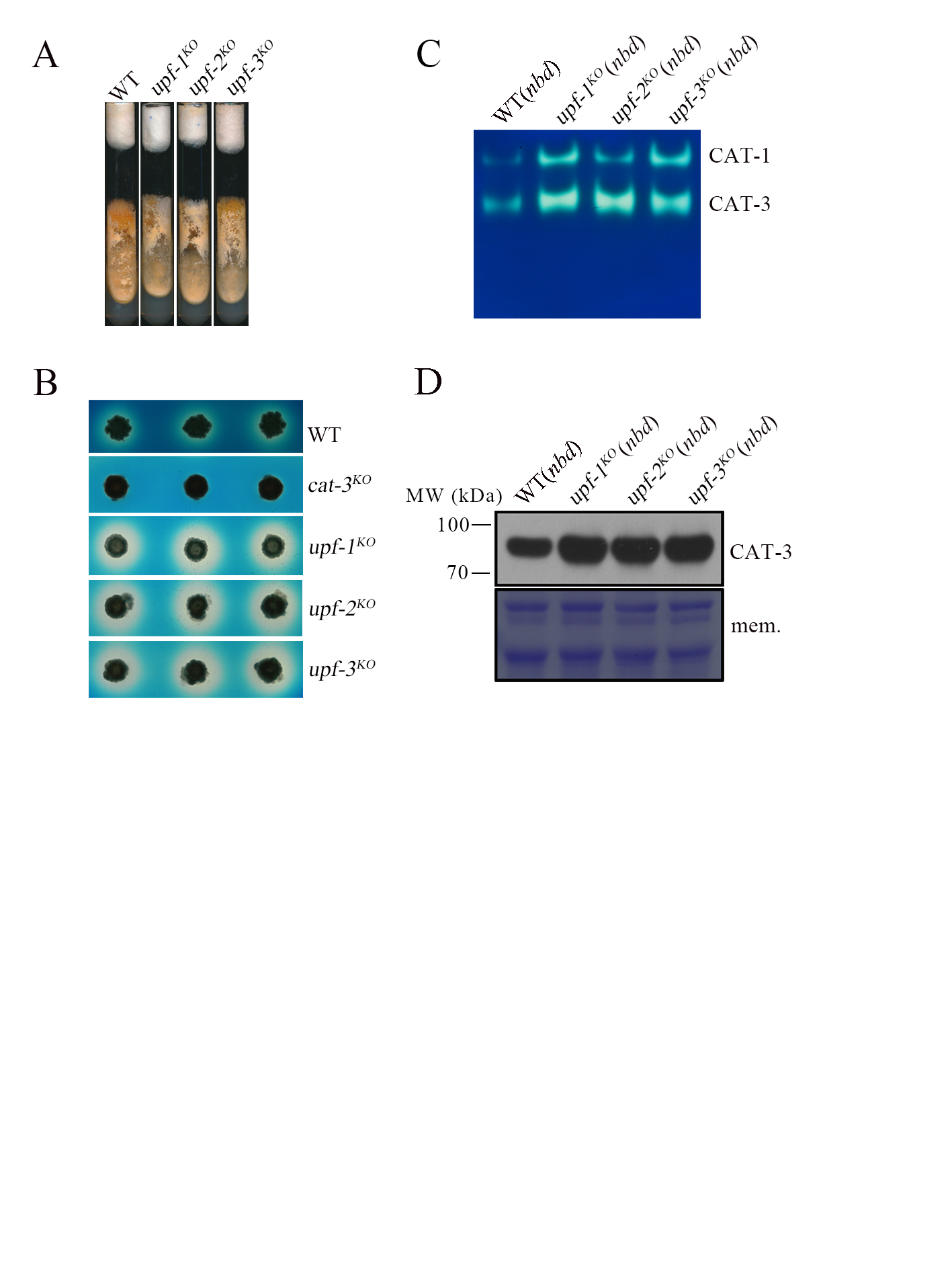

Supplement: S1 Fig — (A) Growth phenotypes of WT, upf-1KO, upf-2KO and upf-3KO strains on slants. (B) Extracellular catalases assay showing the extracellular levels of CAT-3 in the WT and upf mutants. Extracellular catalases were visualized as a transparent circle (halo) where H2O2 was decomposed by catalases. WT clones were stained on day 3, and upf mutants clones were stained on day 4 when they reached the same size as WT clones. (C) In-gel assays showing the CAT-3 activities of WT (FGSC 4200, nbd), upf-1KO (nbd), upf-2KO (nbd) and upf-3KO (nbd) strains. (D) Western blot showing the levels of CAT-3 protein in WT (FGSC 4200, nbd), upf-1KO (nbd), upf-2KO (nbd) and upf-3KO (nbd) strains. The membrane stained by Coomassie blue represented the total proteins in each sample and served as the loading control. (TIF) [file pgen.1010985.s001.tif]

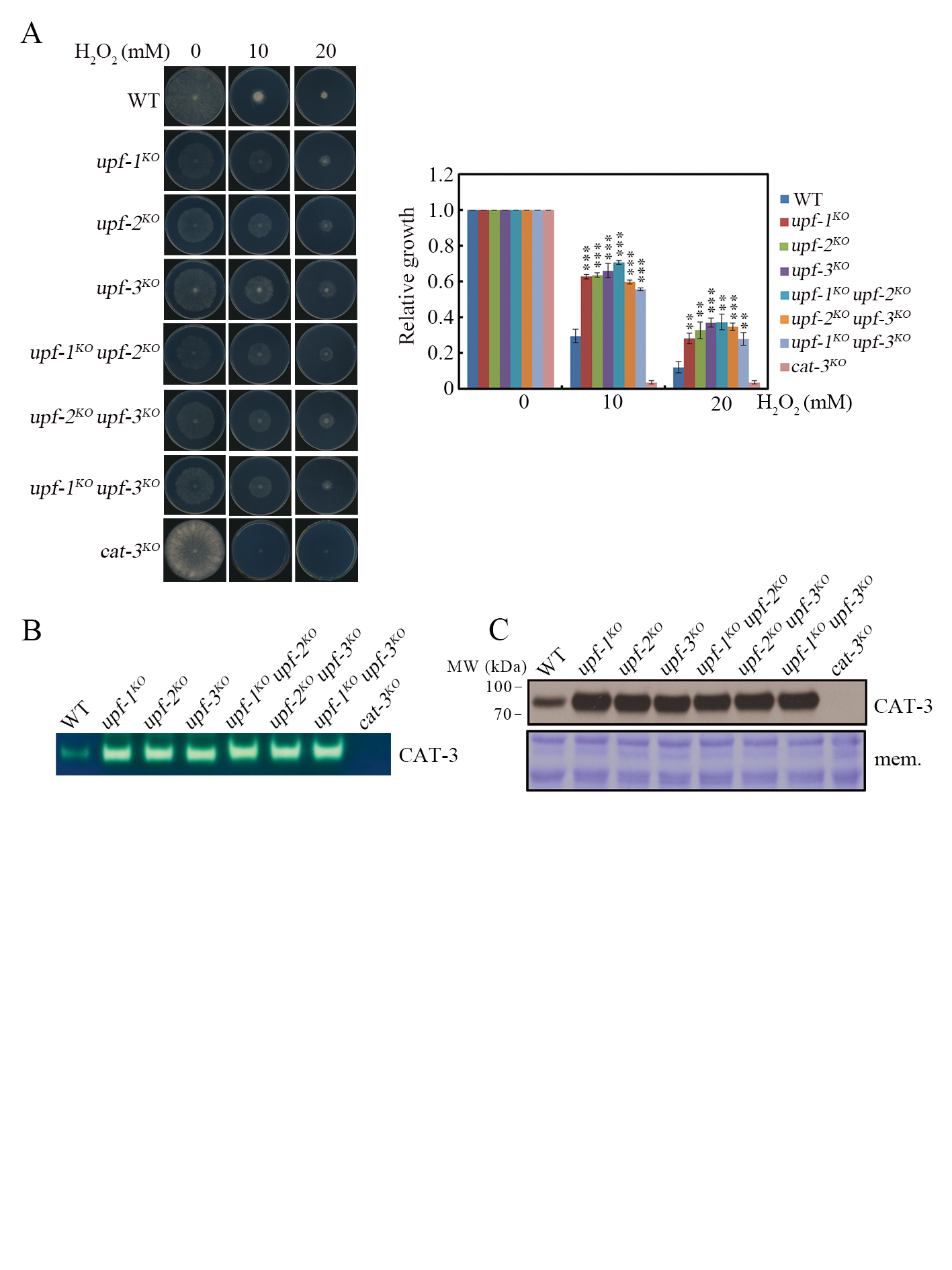

Supplement: S2 Fig — (A) Plate assays analyzing mycelial growth (left) and relative growth statistics (right) of the WT, upf-1KO, upf-2KO, upf-3KO strains and upf-1KO upf-2KO, upf-2KO upf-3KO, upf-1KO upf-3KO double mutants under different H2O2 concentrations. (B) In-gel assays showing the CAT-3 activities of WT, upf-1KO, upf-2KO, upf-3KO strains and upf-1KO upf-2KO, upf-2KO upf-3KO, upf-1KO upf-3KO double mutants. (C) Western blot showing the levels of CAT-3 protein in WT, upf-1KO, upf-2KO, upf-3KO strains and upf-1KO upf-2KO, upf-2KO upf-3KO, upf-1KO upf-3KO double mutants. The membrane stained by Coomassie blue represented the total protein in each sample and served as the loading control. Error bars indicate S.D. (n = 3). *P < 0.05; **P < 0.01; ***P < 0.001. The cat-3KO strain was used as the negative control in (A) (B) (C). (TIF) [file pgen.1010985.s002.tif]

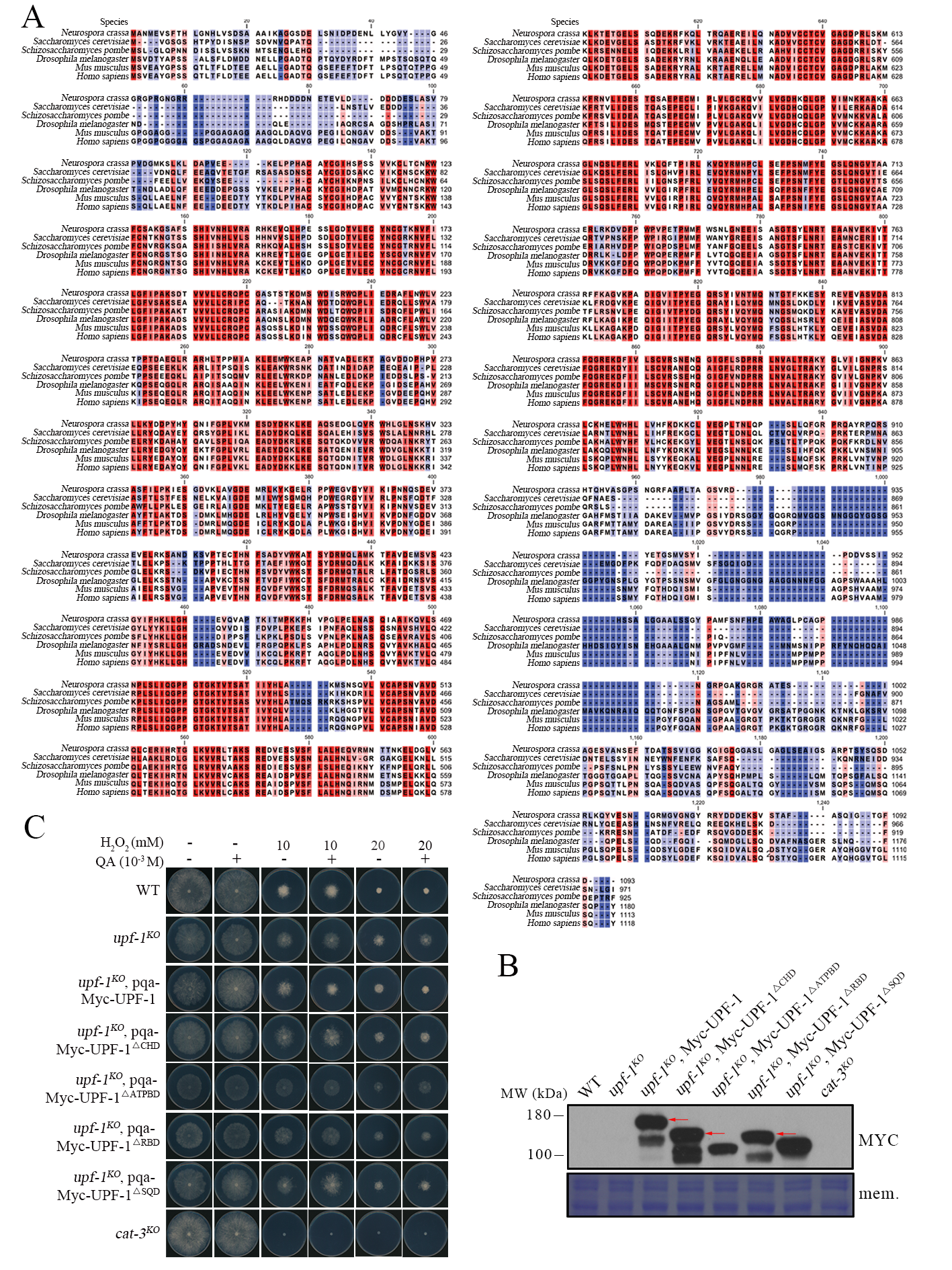

Supplement: S3 Fig — (A) Amino acid sequence alignment of Neurospora crassa UPF-1 protein with its homologous proteins in Schizosaccharomyces pombe, Saccharomyces cerevisiae, Drosophila melanogaster, Mus musculus and Homo sapiens. (B) Western blot showing the protein levels of Myc-UPF-1 in the different deletion strains across UPF-1 coding region at exogenous locus. The arrow shows the specific band. (C) Plate assays analyzing mycelial growth of the different deletion strains across UPF1 coding domain at exogenous locus driven by qa-2 promoter under 0, 10, 20 mM H2O2. The cat-3KO strain was used as the negative control. Quinic acid (QA) was used to induce the qa-2 promoter. (TIF) [file pgen.1010985.s003.tif]

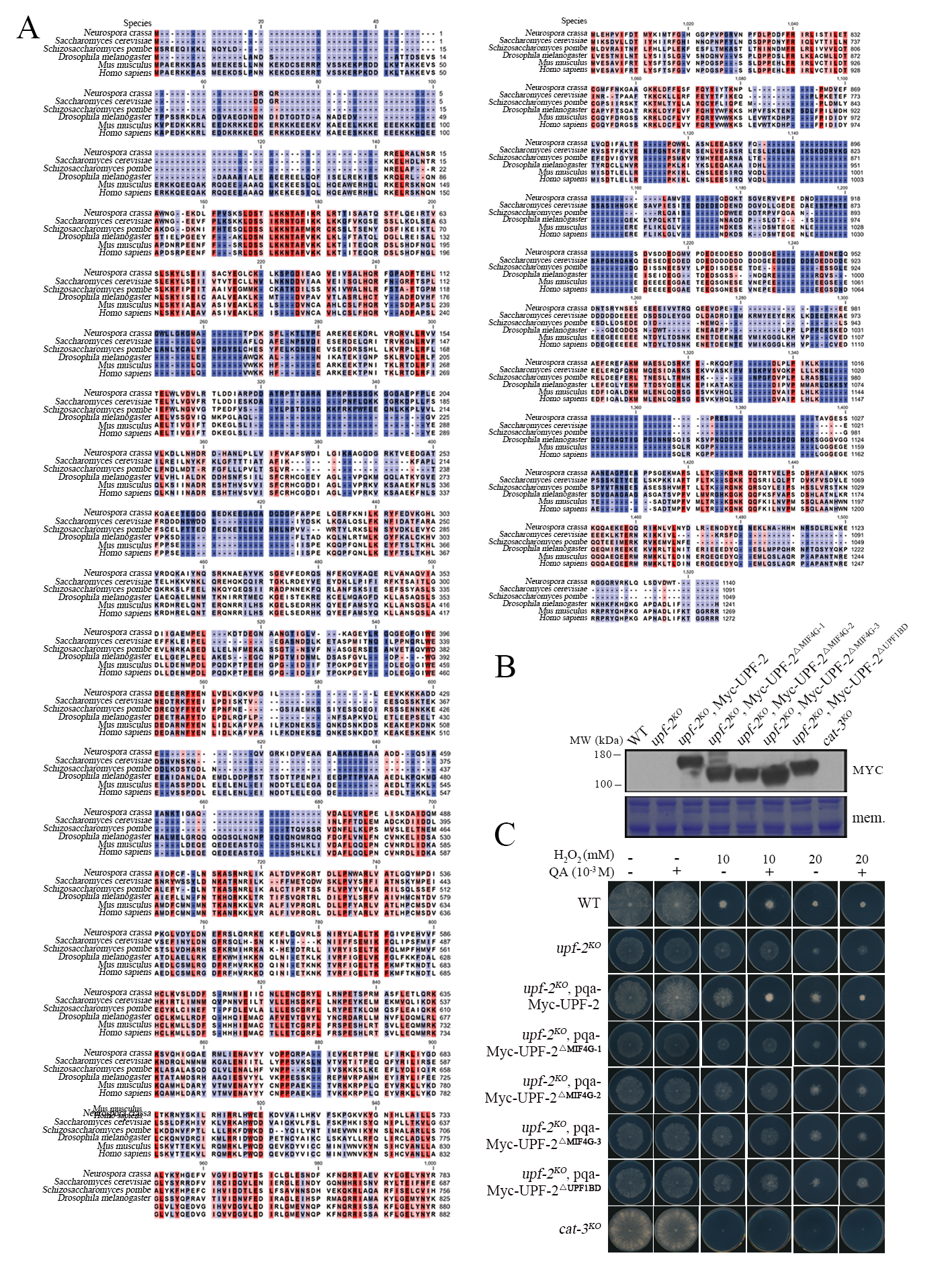

Supplement: S4 Fig — (A) Amino acid sequence alignment of Neurospora crassa UPF-2 protein with its homologous proteins in Schizosaccharomyces pombe, Saccharomyces cerevisiae, Drosophila melanogaster, Mus musculus and Homo sapiens. (B) Western blot showing the protein levels of Myc-UPF-2 in the different deletion strains across UPF-2 coding region at exogenous locus. (C) Plate assays analyzing mycelial growth of the different deletion strains across UPF-2 coding domain at exogenous locus driven by qa-2 promoter under 0, 10, 20 mM H2O2. The cat-3KO strain was used as the negative control. Quinic acid (QA) was used to induce the qa-2 promoter. (TIF) [file pgen.1010985.s004.tif]

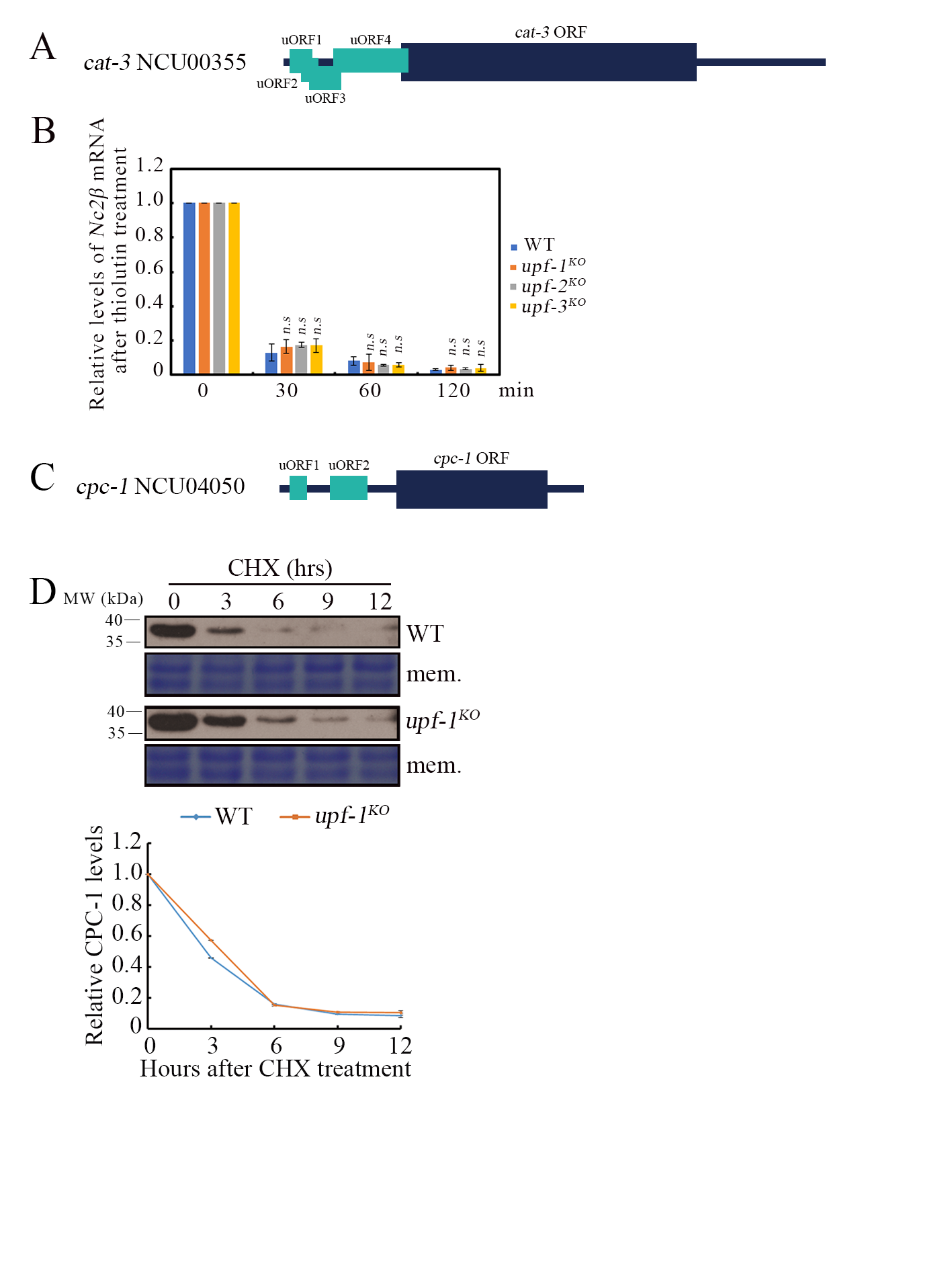

Supplement: S5 Fig — (A) Putative NMD-inducing features (uORFs) in cat-3 transcript. (B) RT-qPCR assays showing the relative degradation ratio of Nc2β mRNA in WT, upf-1KO, upf-2KO and upf-3KO strains after the addition of thiolutin. Error bars indicate S.D. (n = 3). *P < 0.05; **P < 0.01; ***P < 0.001. Unpaired Student’s t test was used. (C) Putative NMD-inducing features (uORFs) in cpc-1 transcript. (D) Western blot showing the degradation of CPC-1 protein in WT and upf-1KO strains after the addition of cycloheximide (CHX). Quantification of the CPC-1 protein level was showed below. The membrane stained by Coomassie blue represented the total protein in each sample and served as the loading control. (TIF) [file pgen.1010985.s005.tif]

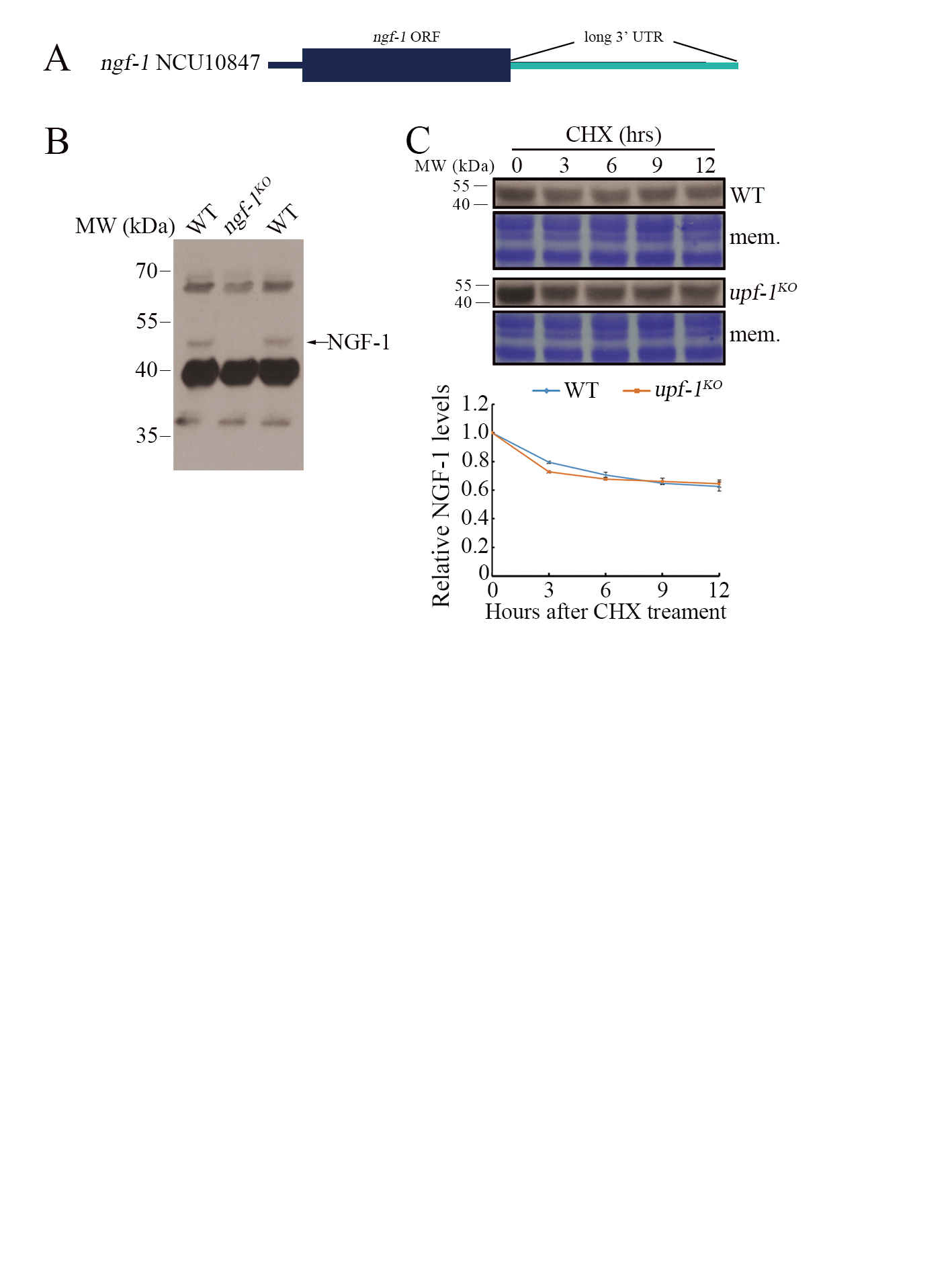

Supplement: S6 Fig — (A) Putative NMD-inducing features (long 3’UTR) in ngf-1 transcript. (B) Immunodetection of NGF-1 in the WT strain using polyclonal antiserum that specifically recognizes endogenous NGF-1 protein. The ngf-1KO strain was used as the negative control. (C) Western blot showing the degradation of NGF-1 protein in WT and upf-1KO strains after the addition of cycloheximide (CHX). Quantification of the NGF-1 protein level was showed below. The membrane stained by Coomassie blue represented the total protein in each sample and served as the loading control. (TIF) [file pgen.1010985.s006.tif]

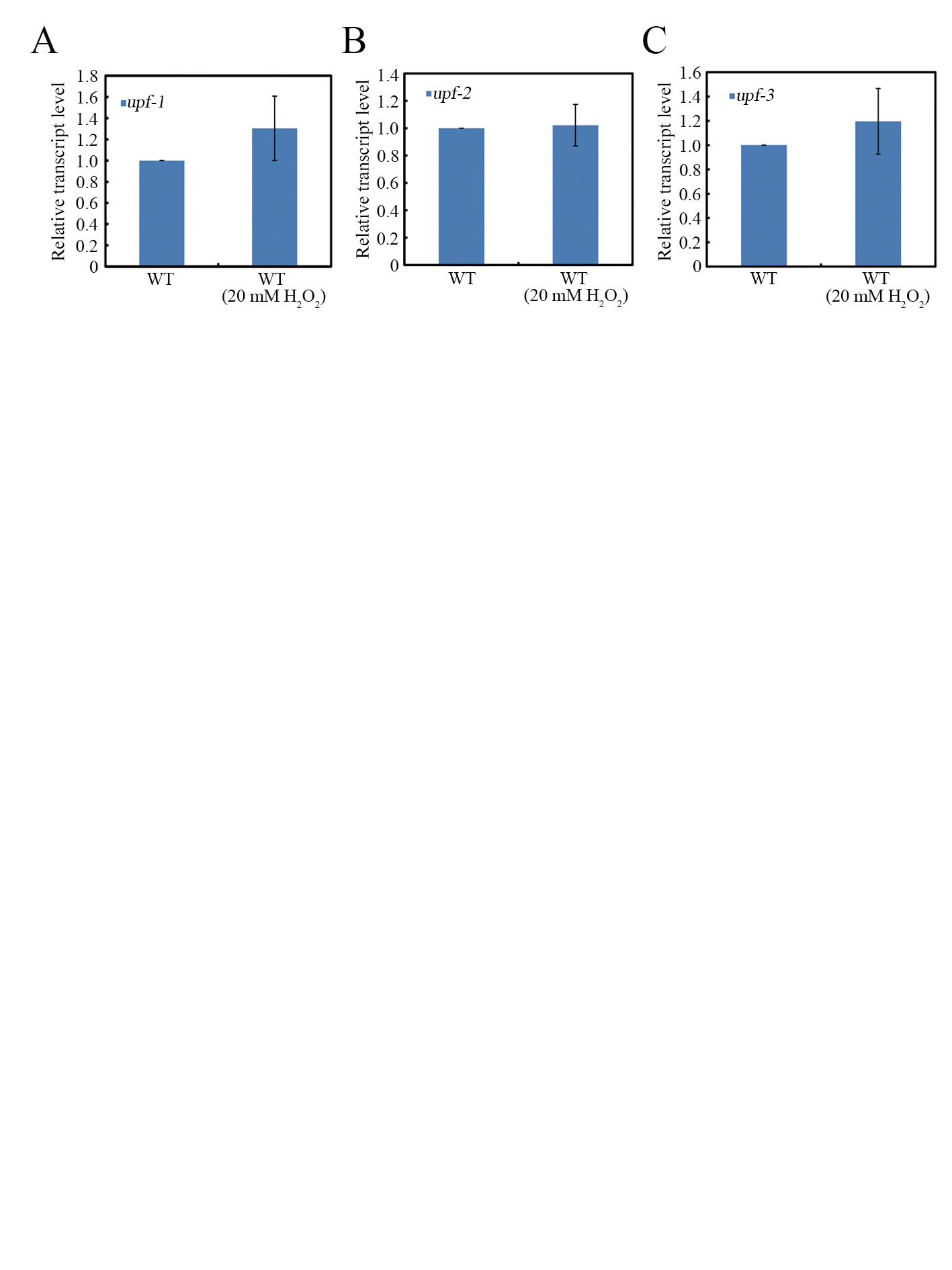

Supplement: S7 Fig — RT-qPCR assays showing the levels of upf-1 (A), upf-2 (B) and upf-3 (C) mRNAs in the WT strain with or without H2O2 treatment. Error bars indicate S.D. (n = 3). N.S. no significance. Unpaired Student’s t test was used. (TIF) [file pgen.1010985.s007.tif]

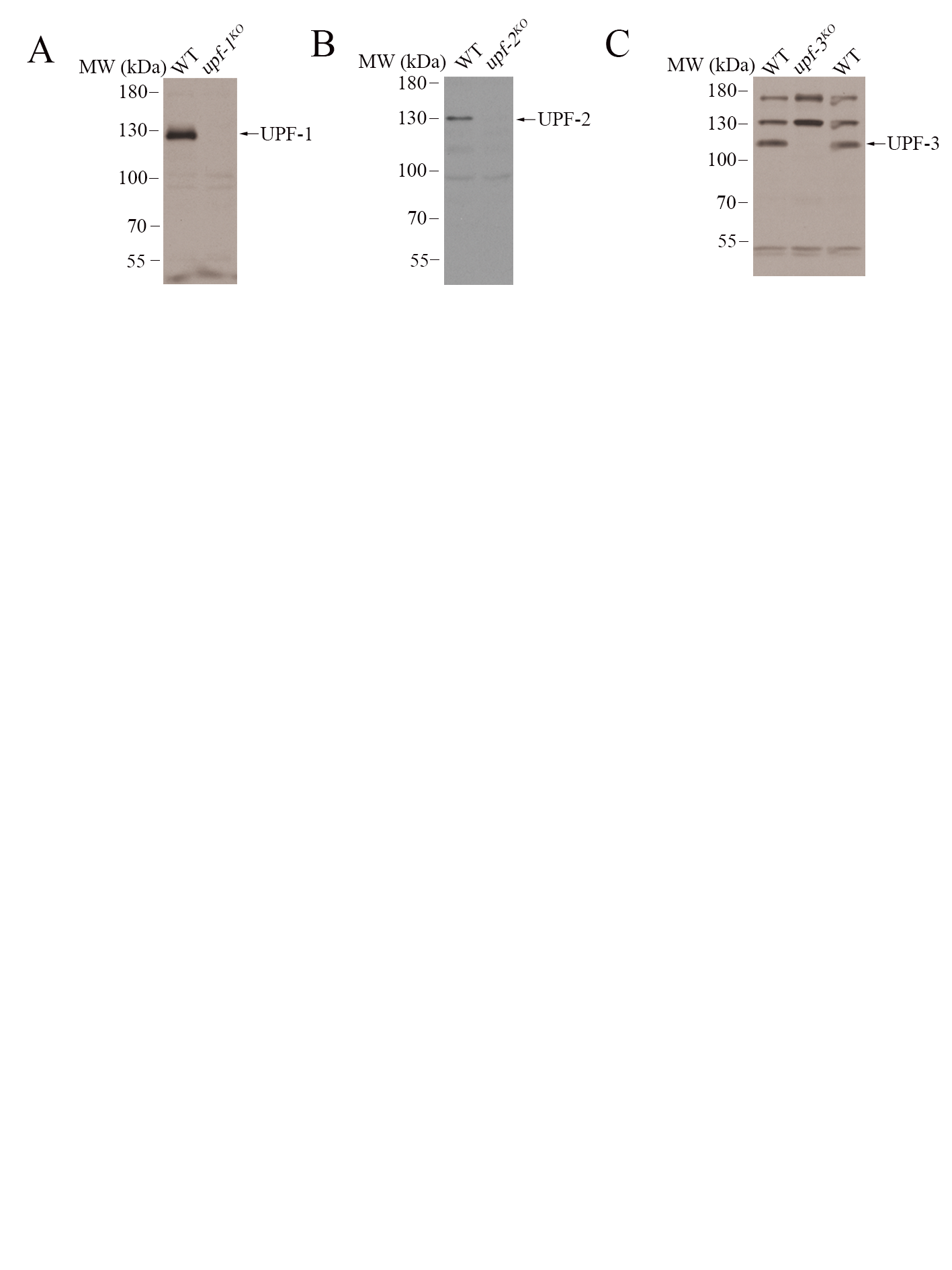

Supplement: S8 Fig — Immunodetection of UPF-1 (A), UPF-2 (B) and UPF-3 (C) in the WT strain using polyclonal antiserum that specifically recognizes endogenous UPF proteins. upf-1KO, upf-2KO and upf-3KO strains were used as the negative control in (A) (B) (C), respectively. (TIF) [file pgen.1010985.s008.tif]

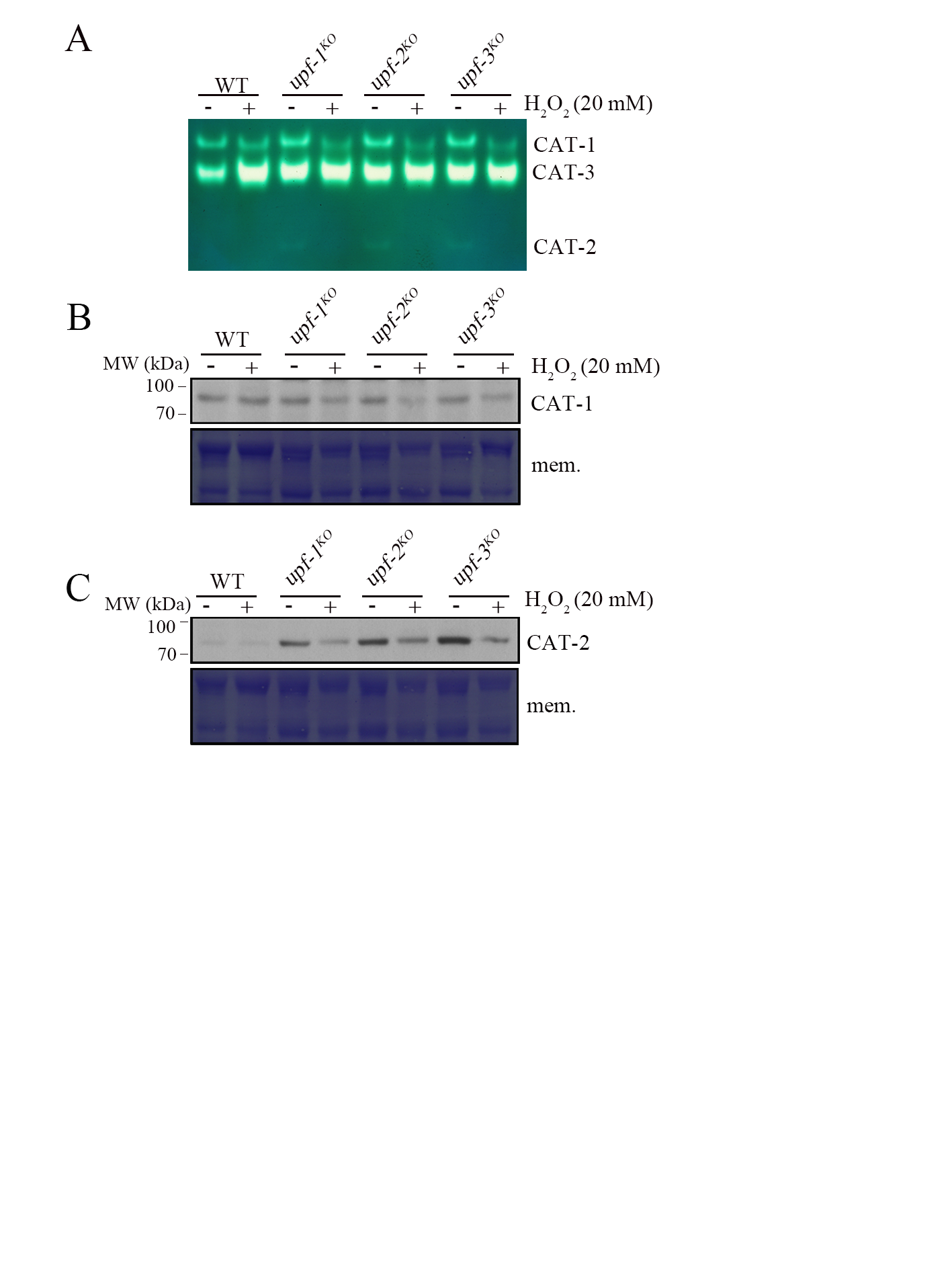

Supplement: S9 Fig — (A) In-gel assays showing the CAT-1 and CAT-2 activities of WT, upf-1KO, upf-2KO and upf-3KO strains with or without H2O2 treatment. (B) and (C) Western blot showing the protein levels of CAT-1 and CAT-2 in WT, upf-1KO, upf-2KO and upf-3KO strains with or without H2O2 treatment. The membrane stained by Coomassie blue represented the total proteins in each sample and served as the loading control. (TIF) [file pgen.1010985.s009.tif]

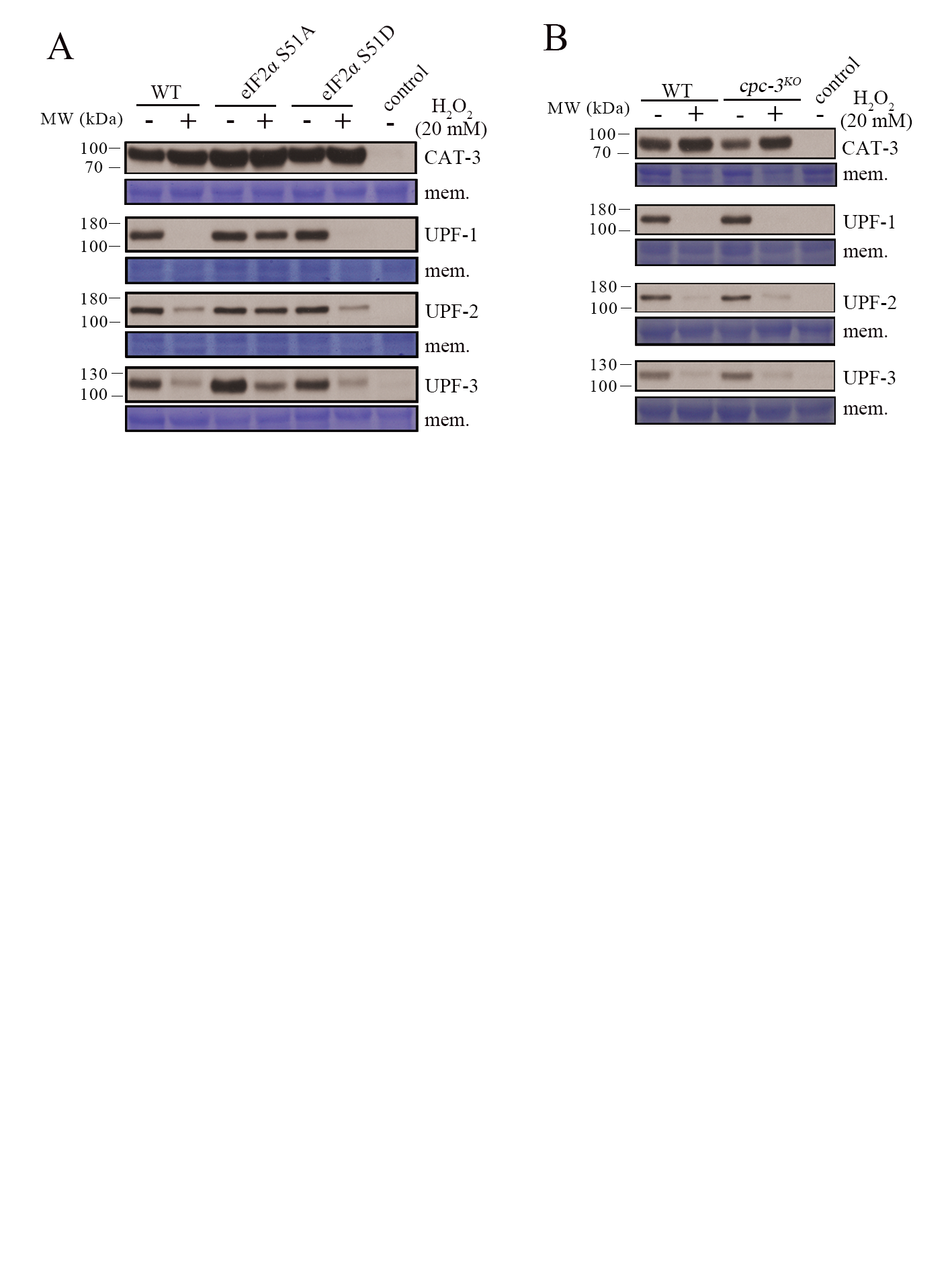

Supplement: S10 Fig — (A) Western blot showing the levels of CAT-3, UPF-1, UPF-2 and UPF-3 proteins in WT, eIF2α S51A and eIF2α S51D strains under 20 mM H2O2. (B) Western blot showing the protein levels of CAT-3, UPF-1, UPF-2 and UPF-3 in WT and cpc-3KO strains under 20 mM H2O2. The corresponding controls indicated cat-3KO, upf-1KO, upf-2KO and upf-3KO strains. The membrane stained by Coomassie blue represented the total protein in each sample and served as the loading control. (TIF) [file pgen.1010985.s010.tif]
